# Supplementary material for: Structural insights into terminal arabinosylation of mycobacterial cell wall arabinan
Source: Nat Commun. 2025 Apr 29;16:3973. doi: 10.1038/s41467-025-58196-5 (PMC12041299; doi:10.1038/s41467-025-58196-5)
Supplement: Supplementary file 1 — Supplementary Information [file 41467_2025_58196_MOESM1_ESM.pdf]

Structural insights into terminal arabinosylation of mycobacterial cell wall arabinan

Yaqi Liu[1], Chelsea M. Brown[2,3], Satchal Erramilli[4], Yi-Chia Su[5], Shih-Yun Guu[5], Po-Sen Tseng[6], Yu-Jen Wang[5], Nam Ha Duong[5,7,8], Piotr Tokarz[4], Brian Kloss[1], Cheng-Ruei Han[5], Hung-Yu Chen[5], José Rodrigues[9], Kay-Hooi Khoo[5,10], Margarida Archer[9], Anthony A. Kossiakoff[4], Todd L. Lowary[5,6,10 *], Phillip J. Stansfeld[2, *], Rie Nygaard[1, 11, *] and Filippo Mancia[1, *]

[1]Department of Physiology and Cellular Biophysics, Columbia University Irving Medical Center, New York, NY, USA.

[2]School of Life Sciences & Department of Chemistry, University of Warwick, Coventry, UK.

[3]Groningen Biomolecular Sciences and Biotechnology Institute and Zernike Institute for Advanced Materials, University of Groningen, Nijenborgh 7, 9747 AG Groningen, The Netherlands.

[4]Department of Biochemistry and Molecular Biophysics, University of Chicago, Chicago, IL, USA.

[5]Institute of Biological Chemistry, Academia Sinica, Nangang, Taipei, Taiwan.

[6]Department of Chemistry, University of Alberta, Edmonton, AB, Canada.

[7]Chemical Biology and Molecular Biophysics, Taiwan International Graduate Program, Academia Sinica, Nangang, Taipei, Taiwan.

[8]Department of Chemistry, National Tsing Hua University, Hsinchu, Taiwan.

[9]Instituto de Tecnologia Química e Biológica António Xavier, Universidade Nova de Lisboa (ITQB-UNL), Oeiras, Portugal.

[10]Institute of Biochemical Sciences, National Taiwan University, Taipei, Taiwan.

[11]Department of Radiation Oncology, Weill Cornell Medicine, New York, NY, USA.

*Correspondence to be addressed to: tlowary@as.edu.tw (T.L.L), Phillip.Stansfeld@warwick.ac.uk (P.S.), rin7007@med.cornell.edu (R.N.), fm123@columbia.edu (F.M.)

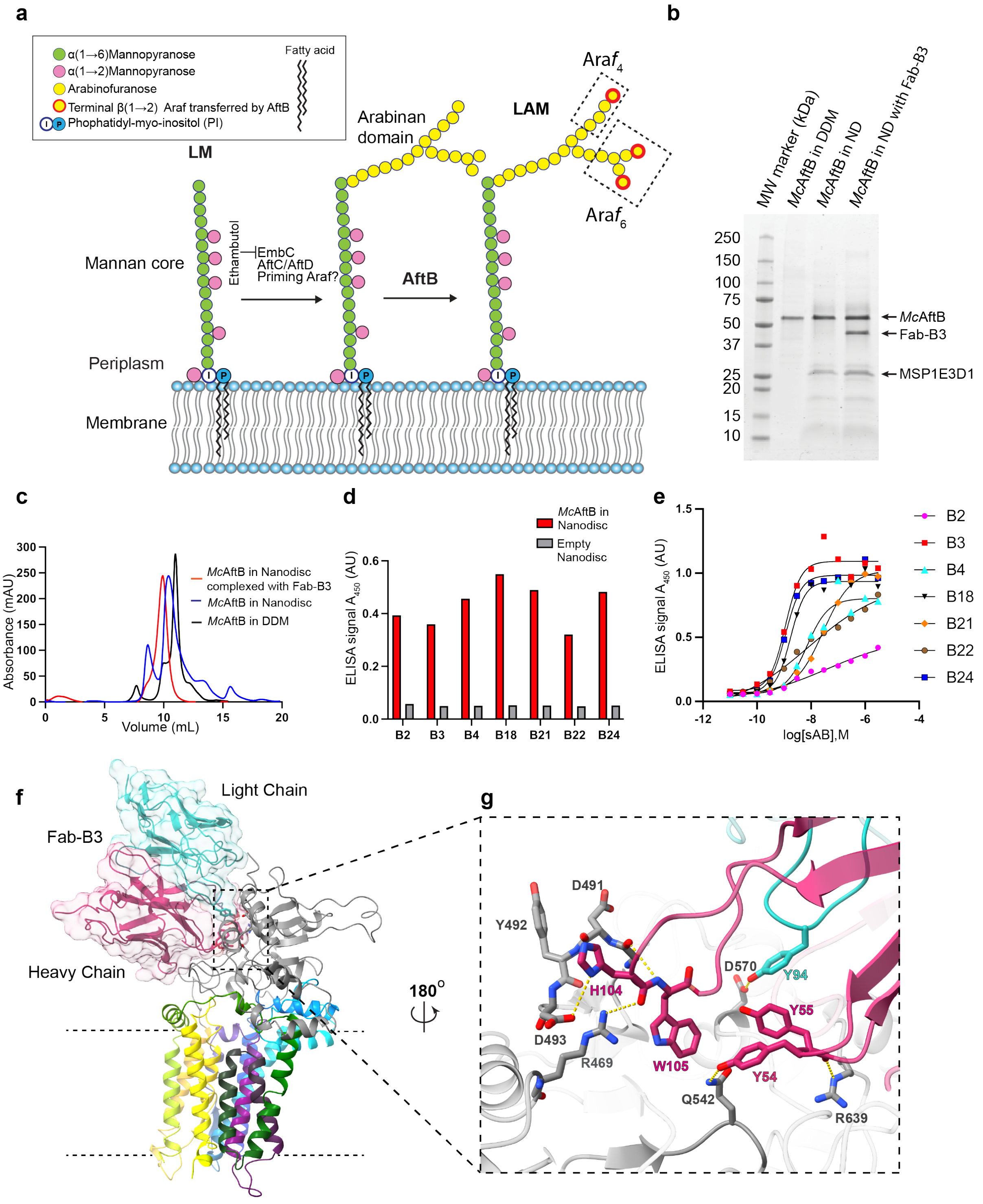

Supplementary Fig. 1 Characterization and structural analysis of AftB.

(a) Schematic representation of biosynthesis of arabinan domain of LAM catalyzed by AraTs, highlighting the role of the terminal arabinose addition catalyzed by AftB. (b) SDS-PAGE gel of AftB purification. The first lane shows AftB purified in DDM, the second lane shows AftB reconstituted into nanodiscs (using MSP1E3D1 and POPG), and the third lane shows AftB reconstituted into nanodiscs (MSP1E3D1 and POPG) with Fab-B3 bound. This experiment was repeated three times with similar results. (c) SEC elution profile of purified AftB in detergent (black), incorporated into a nanodisc (blue), and incorporated into a nanodisc with Fab-B3 bound (red). (d) Single-point ELISA measuring the binding of phage-displayed sABs to AftB in MSP1E3D1 nanodiscs (red), empty nanodiscs (light grey). ELISA signal was measured at 450 nm absorbance. Data are presented as a bar chart from a single experiment, where the bar height corresponds to the measured signal intensity for each Fab clone. (e) Multi-point sAB ELISA: EC50 estimation for purified sAB binding to AftB incorporated into MSP1E3D1 nanodiscs, showing binding of B2, B3, B4, B18, B21, B22, B24. Data from a single experiment is shown. (f) AftB-Fab-B3 complex structure shown in ribbon with AftB's TM domain in rainbow colors and PD in grey. The Fab's light chain is depicted in teal, and the heavy chain in pink. (g) Magnified view of the interface between AftB and Fab-B3, highlighting the key interactions.

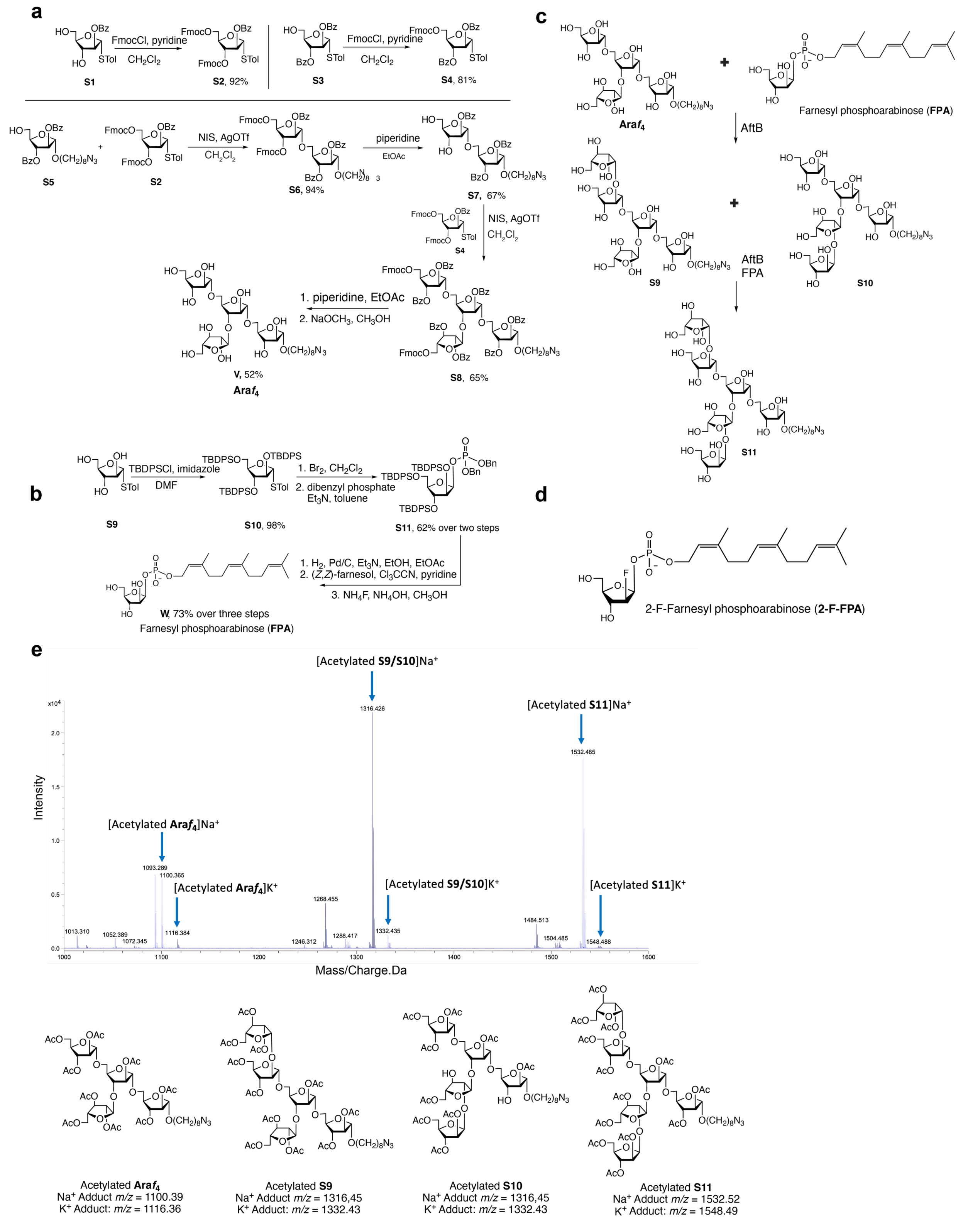

Supplementary Fig. 2 Substrate synthesis and AftB enzymatic activity.

(a) Synthesis of Ara*f*4 and (b): Synthesis of FPA (c) AftB-catalyzed arabinofuranosylation of acceptor Ara*f*4. The first formed products are pentasaccharide S9 and S10 and then hexasaccharide S11. (d) Chemical structure of 2F-FPA. (e) Assay mixtures were produced by including acceptor Ara*f*4, FPA, and *E. coli* membranes containing recombinant wild type AftB, followed by passage through an ion exchange cartridge and acetylation of the eluant.

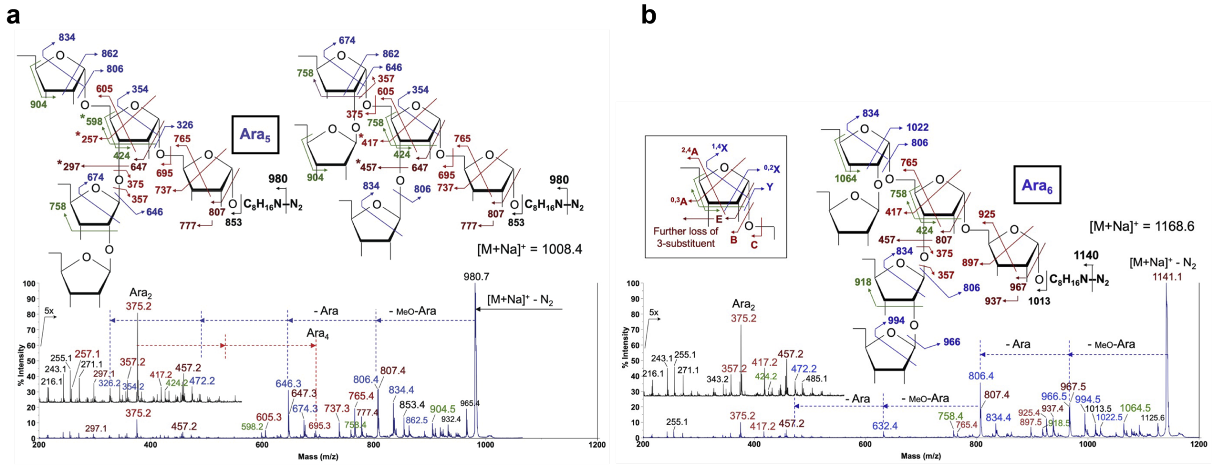

Supplementary Figure 3. Confirmation of linkage formation assessed by MALDI mass spectrometry. MS/MS analysis of the products confirmed the (1→2)-linkages in the pentasaccharide (a) and hexasaccharide products (b) derived from the tetrasaccharide acceptor.

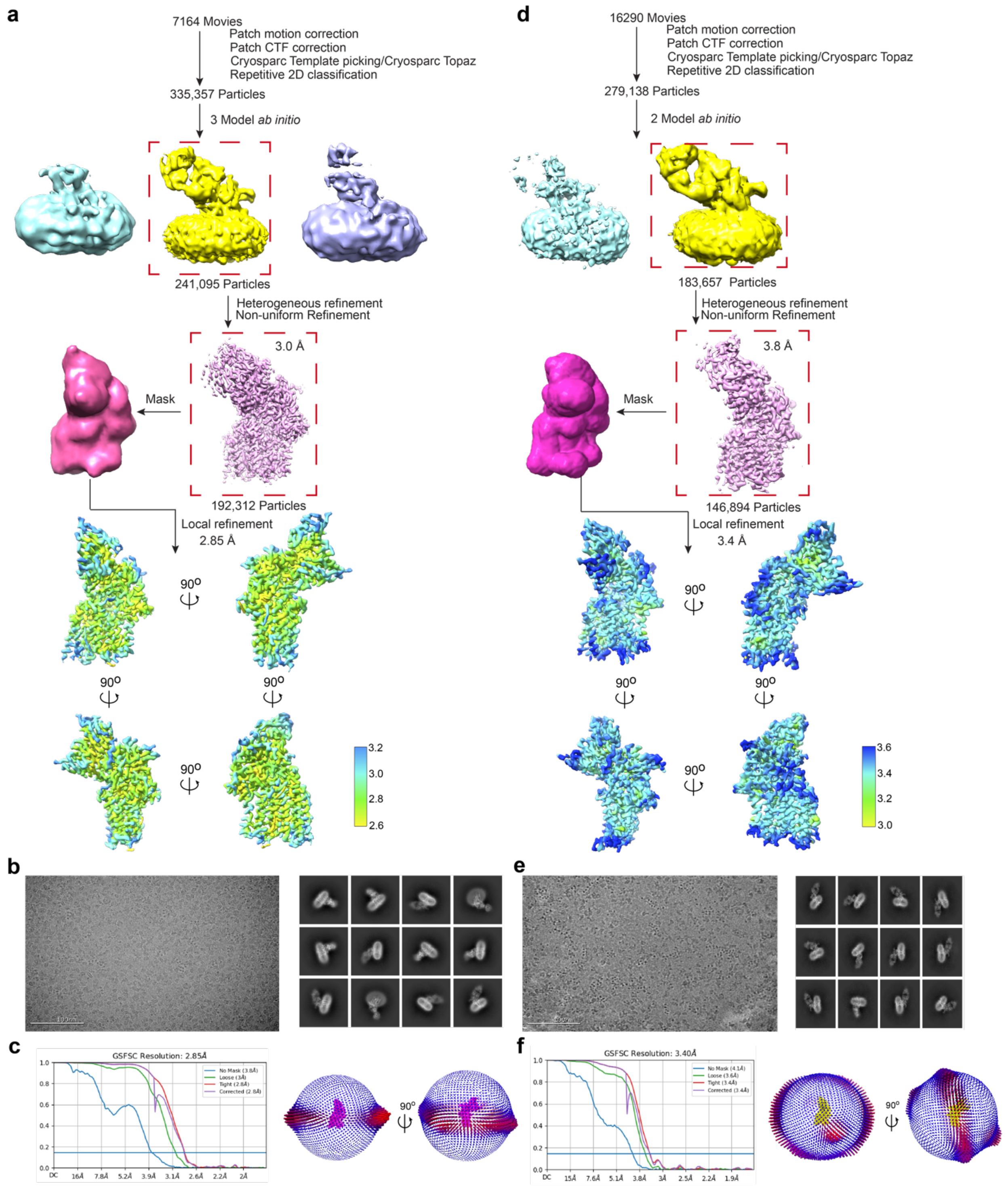

Supplementary Fig. 4 Cryo-EM analysis of *Mc*AftB.

(a) Schematic depiction of the cryo-EM data processing and structure determination for nanodisc-reconstituted apo *M.chubuense* AftB complexed with Fab-B3 using cryoSPARC. (b) Representative micrograph and representative 2D classes. (c) Top: Fourier shell correlation (FSC) curves of 3D reconstruction. Bottom: Euler angle distribution of particles used in the final 3D reconstruction. Final map shown in magenta. Each orientation is represented by a cylinder, with each cylinder's height and color (from blue to red) proportional to the number of particles for that specific direction. (d) Schematic depiction of the cryo-EM data processing and structure determination for 2F-FPA bound AftB complexed with Fab-B3 using cryoSPARC. (e) Representative micrograph and representative 2D class. (f) Top: Fourier shell correlation (FSC) curves of 3D reconstruction. Bottom: Euler angle distribution of particles used in the final 3D reconstruction. Final map is shown in magenta. Each orientation is represented by a cylinder, with each cylinder's height and color (from blue to red) proportional to the number of particles for that specific direction.

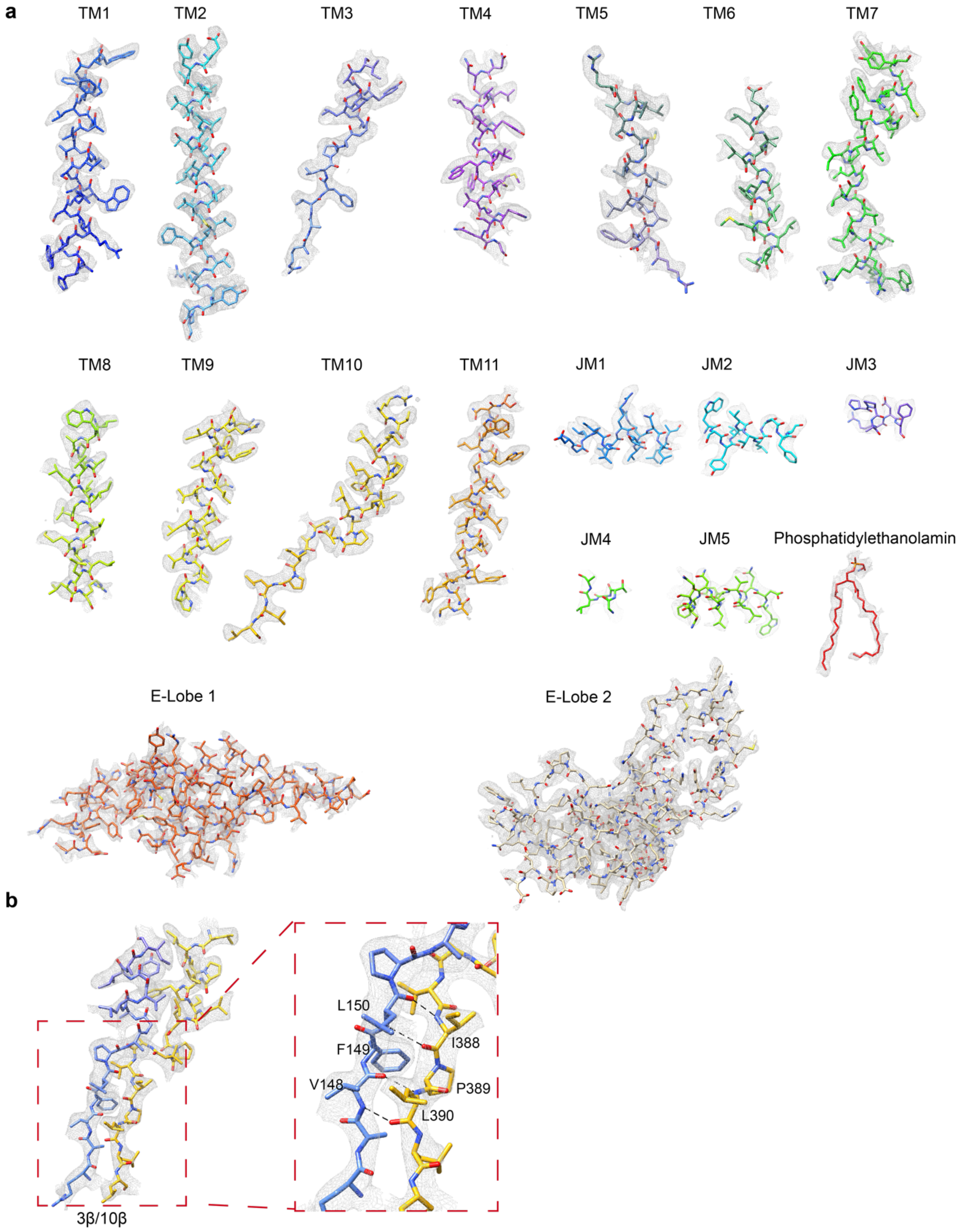

Supplementary Fig. 5 EM Density of apo *Mc*AftB.

(a) The atomic model of the structure of *Mc*AftB is colored in rainbow and rendered as a cartoon, with the side chains rendered as sticks. The map density is displayed as a mesh. The map are contoured at a level of 3.0 (b) Density for 3β-10β, hydrogen bond is indicated with dashed lines.

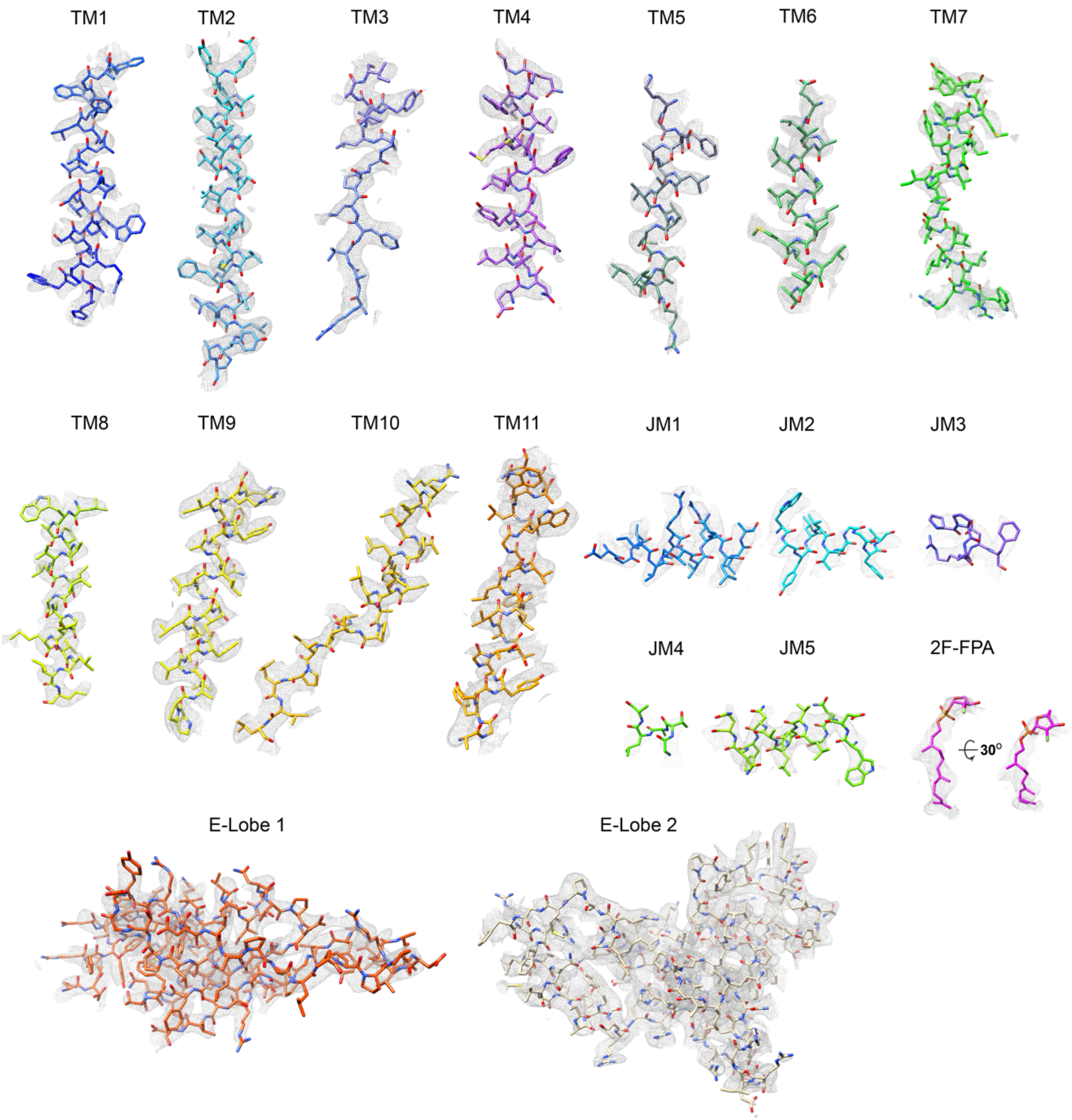

Supplementary Fig. 6 EM Density of 2F-FPA bound *Mc*AftB.

The atomic model of the structure of AftB is colored in rainbow and rendered as a cartoon, with the side chains depicted as sticks. The map is contoured at a level of 1.8. The map density is displayed as a mesh. Density for 2F-FPA is displayed as mesh shown in two different views.

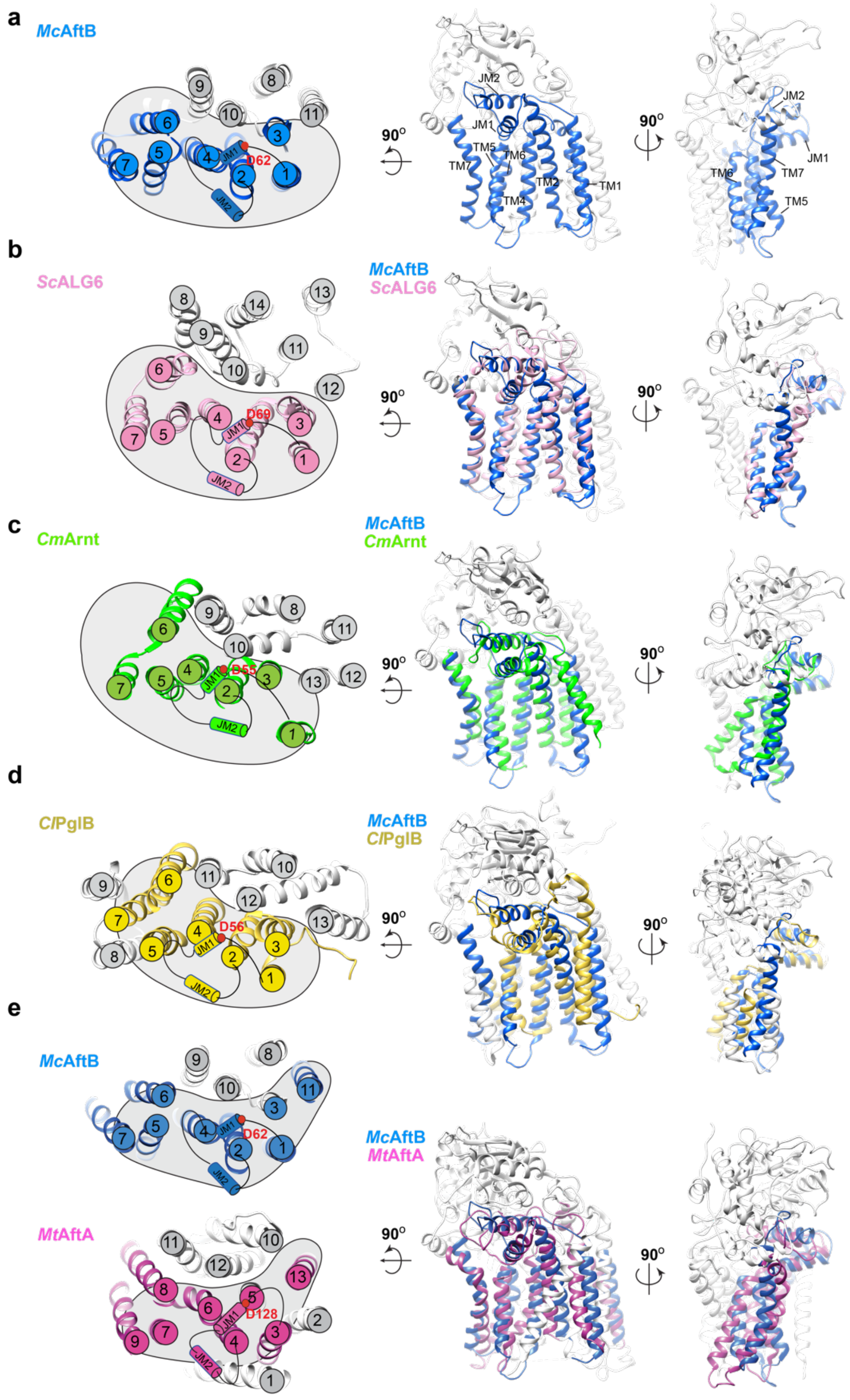

Supplementary Fig. 7 Overall structure and comparison of *Mc*AftB with other GT-C fold glycosyltransferases.

(a) Left, Top-down view of the TM domain of *Mc*AftB, highlighting the TM helices' spatial arrangement, with the first seven conserved helices colored in cyan, others in grey. TM helices are numbered with circles, JM1 and JM2 are represented as cylinders, and the catalytic residue is indicated by a red dot, in reference to Figure 1D. Middle and Right, two orthogonal views of *Mc*AftB. (b-e) Superimposition of *Mc*AftB with characteristic GT-C proteins from diverse species – *S. cerevisiae* ALG6 (PDB: 6SNH), *C. metallidurans* Arnt (PDB: 5F15), *C. lari* PglB (PDB: 5OGL), and *M. tuberculosis* AftA (PDB: 8IF8) – illustrated in three distinct orientations, as presented in panel a.

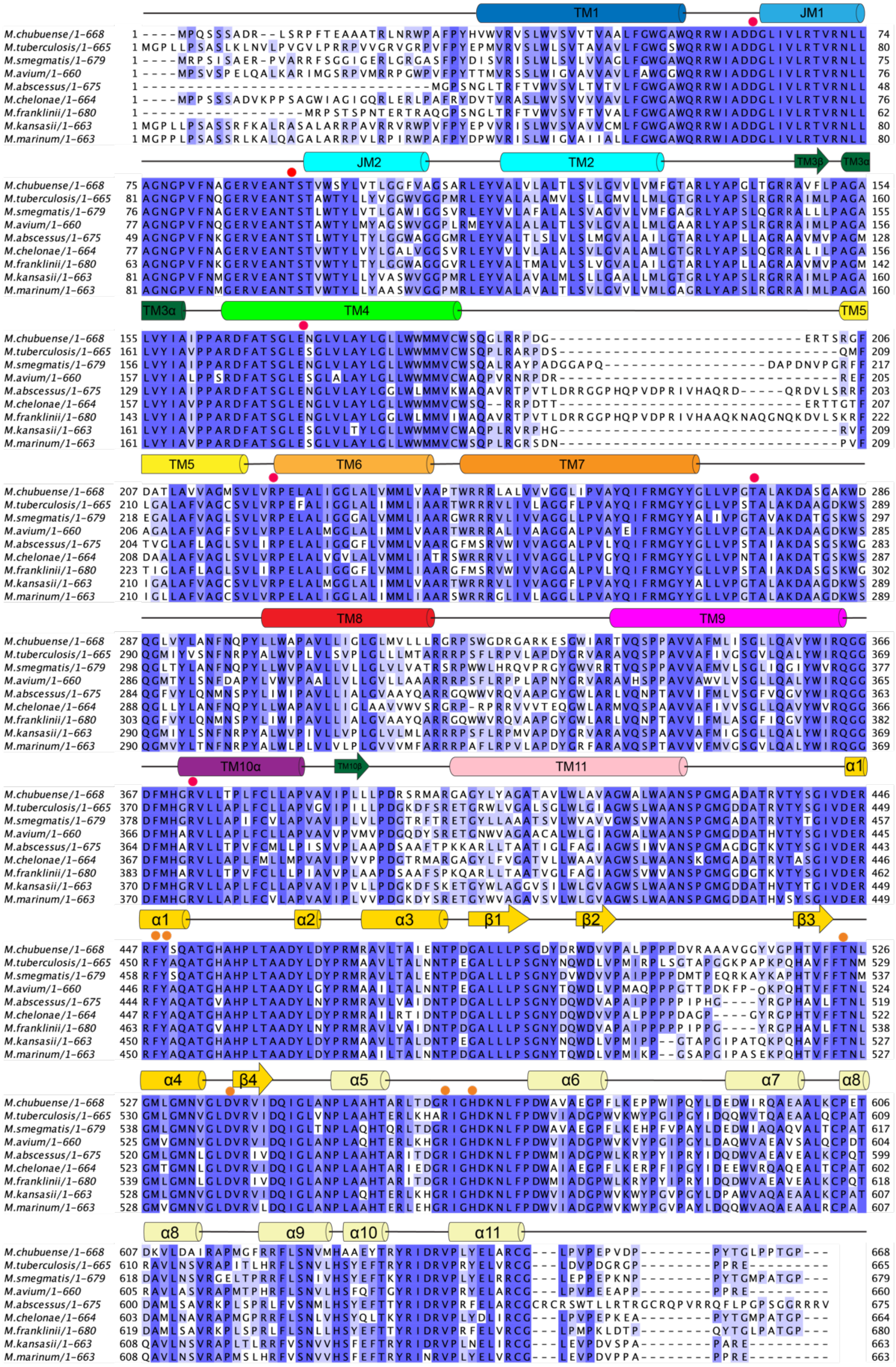

Supplementary Fig. 8 Amino acid sequence conservation of AftB across mycobacterium species.

Sequence alignment of AftB from *M. chubuense, M. tuberculosis, M. smegmatis, M. avium, M. abscessus, M. chelonae, M. franklinii, M. kansasii,* and *M. marinum*. The secondary structure elements, based on *M. chubuense* AftB, are indicated above the sequences. The structurally conserved and catalytically essential residue D62 and all other key residues interacting with 2F-FPA in the structure are marked with a red dot. Residues predicted to be important for acceptor substrate accommodation are indicated with an orange dot.

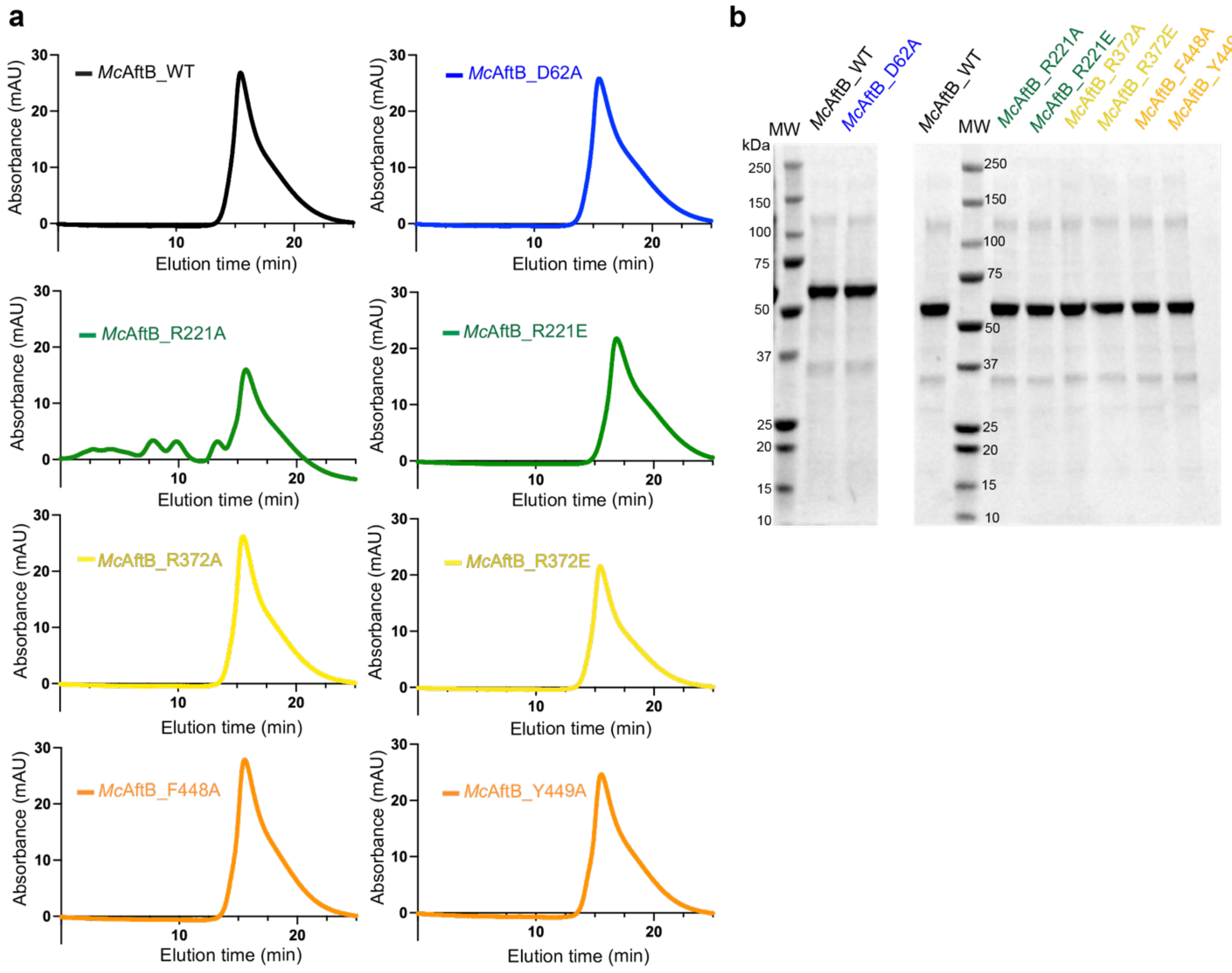

Supplementary Figure 9. Biochemical characterization of wild-type and mutant *Mc*AftB proteins.

(a) Size exclusion chromatography profiles of wild-type *Mc*AftB and mutants (D62A, R221A, R221E, R372A, R372E, F488A, F489A), showing comparable elution volumes that indicate proper protein folding. (b) SDS-PAGE analysis of the peak fractions from each size exclusion chromatography run, demonstrating similar expression levels of wild-type and mutant *Mc*AftB proteins. All the constructs' labels and their corresponding chromatogram traces are colored according to the rainbow scheme used in Figure 3a. This analysis represents a single experimental run. Source data are provided as a Source Data file.

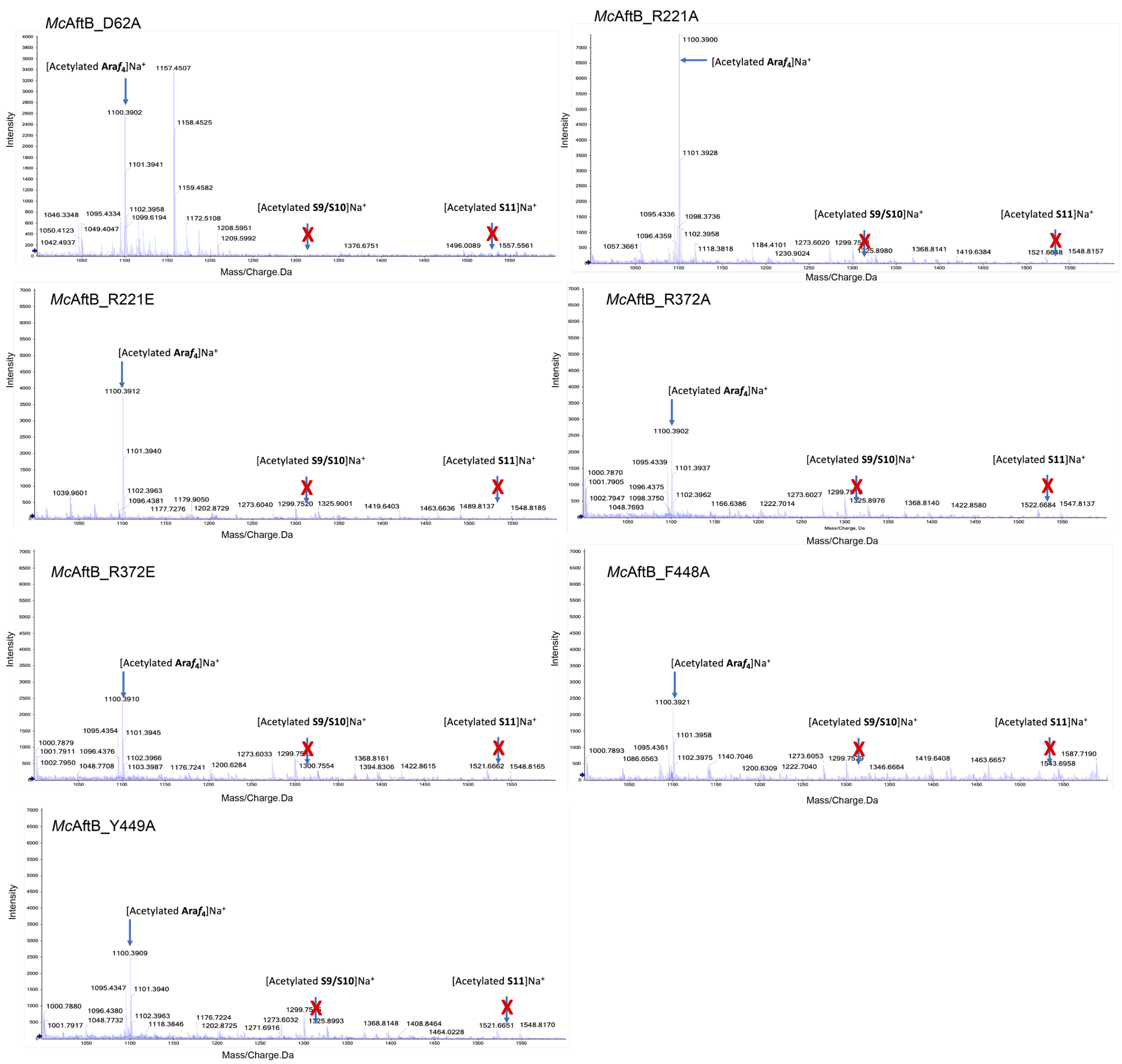

Supplementary Figure 10. Enzymatic activity of *Mc*AftB mutants.

Spectra for wild-type *Mc*AftB and mutants D62A, R221A, R221E, R372A, R372E, F448A, and F449A are shown. Samples were prepared by incubating substrates with recombinant *Mc*AftB or mutants and analyzing the reaction products via MALDI mass spectrometry. No product formation was detected for the mutants, indicating a lack of enzymatic activity. The results highlight the

critical roles of the tested residues in the catalytic function of *Mc*AftB. Mass-to-charge ratios (m/z) and signal intensities are displayed for each spectrum.

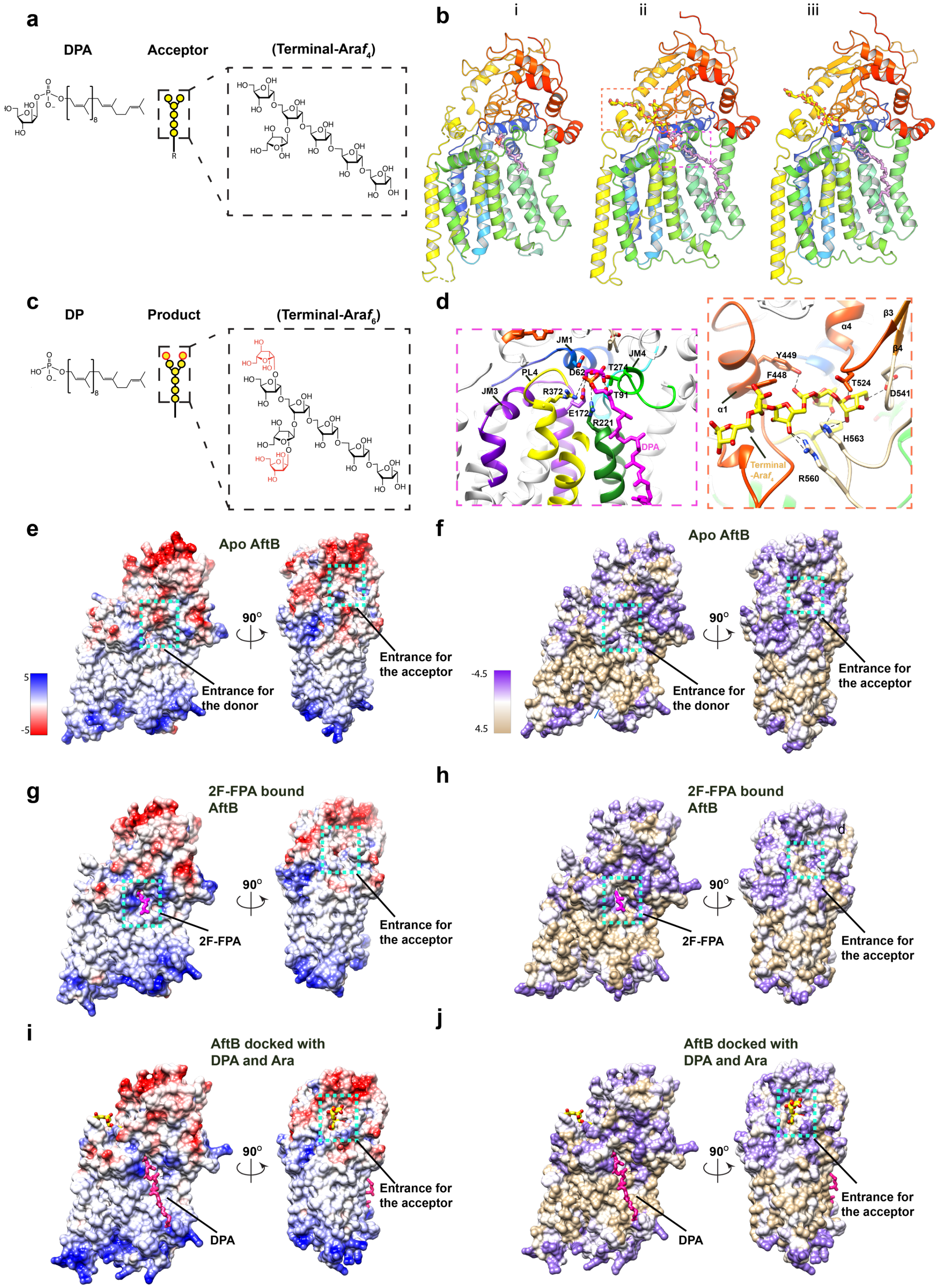

Supplementary Fig. 11 Structural analysis and substrate binding models of AftB.

(a) Donor substrate (DPA) and acceptor mimic (b) used in CG-MD simulations. (c) Computational models predicted by RoseTTAFold All-Atom docking[1]: (i) Structure of the apo *Mc*AftB solved by cryo-EM in this study. (ii) Computational model of AftB in complex with DPA and terminal-Araf_4, predicted by RoseTTAFold docking. (iii) Computational model of AftB in complex with DP and terminal-Araf_6, predicted by RoseTTAFold docking. (d) Detailed views of the substrate binding modes for the RoseTTAFold model shown in c (ii): Left: A magnified view of the binding mode for DPA in AftB showing key interacting residues. The catalytic residue D62 is positioned near the anomeric carbon of DPA. Coordinating residues for the phosphate group (R372, R221, T91, T243) are highlighted. Right: Magnified view of the proposed binding mode for the acceptor substrate in the PD of AftB. Key interacting residues are labeled. (e-f) Surface representation of apo *Mc*AftB colored by electrostatic potential (e) and hydrophobicity (f), shown in different orientations. Putative entrances for donor and acceptor substrates are highlighted with cyan dots. (g-h) Surface representation of 2F-FPA bound *Mc*AftB colored by electrostatic potential (g) and hydrophobicity (h), shown in different orientations. The 2F-FPA bound cavity and putative entrance for acceptor substrates are highlighted with cyan dots. (i-j) Surface representation of *Mc*AftB docked with DPA and simplified acceptor substrate terminal-Araf_4, colored by electrostatic potential (i) and hydrophobicity (j), shown in different orientations. The cavities bound with DPA and terminal-Araf_4 are highlighted with cyan dots. All the electrostatic potential was rendered on a range of ±5 kBT/e. All the hydrophobicity was colored using the kdHydrophobicity scale[2].

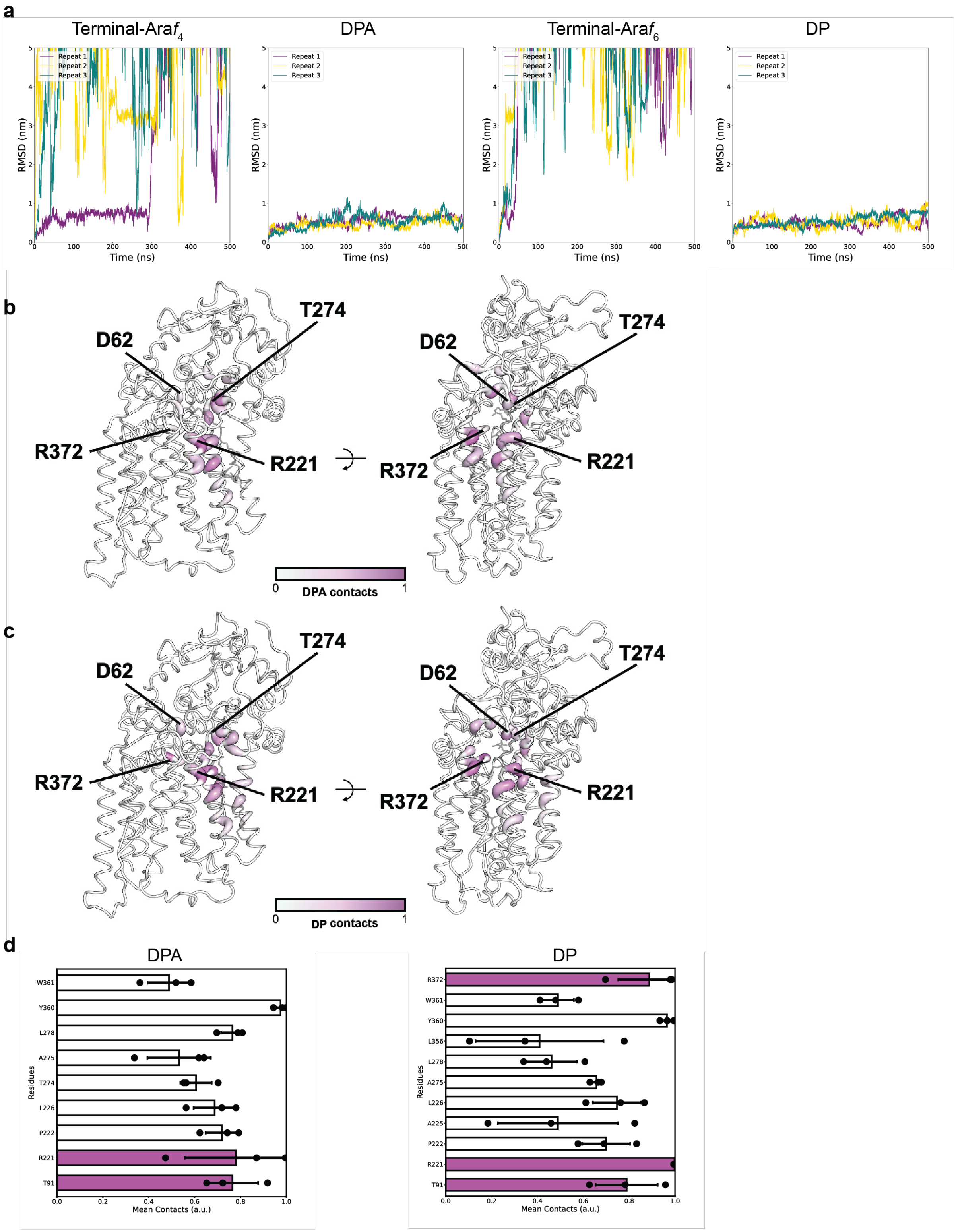

Supplementary Fig. 12 Molecular dynamics analysis of AftB-substrate interactions.

(a) The root mean squared deviation (RMSD) of DPA/DP and terminal-Araf_4/ terminal-Araf_6 with AftB. Each line represents an independent repeat. (b) Areas of AftB in contact with DPA in simulations. The darker the color, the more contacts throughout all simulations. The thickness of the cartoon also represents the number of contacts, where a contact value of 1 would represent contact with the ligand for the entire simulation. Key residue positions have been highlighted. (c) shows the same as (b), but for DP. (d) Contact graphs between AftB and DPA/DP. The mean contact value of three simulations is shown, with the error bar representing standard error. Residues that are in contact with 2F-FPA are highlighted. A contact value of 1 would represent contact with the ligand for the entire simulation, residues with contact values below 0.4 have been omitted for clarity.

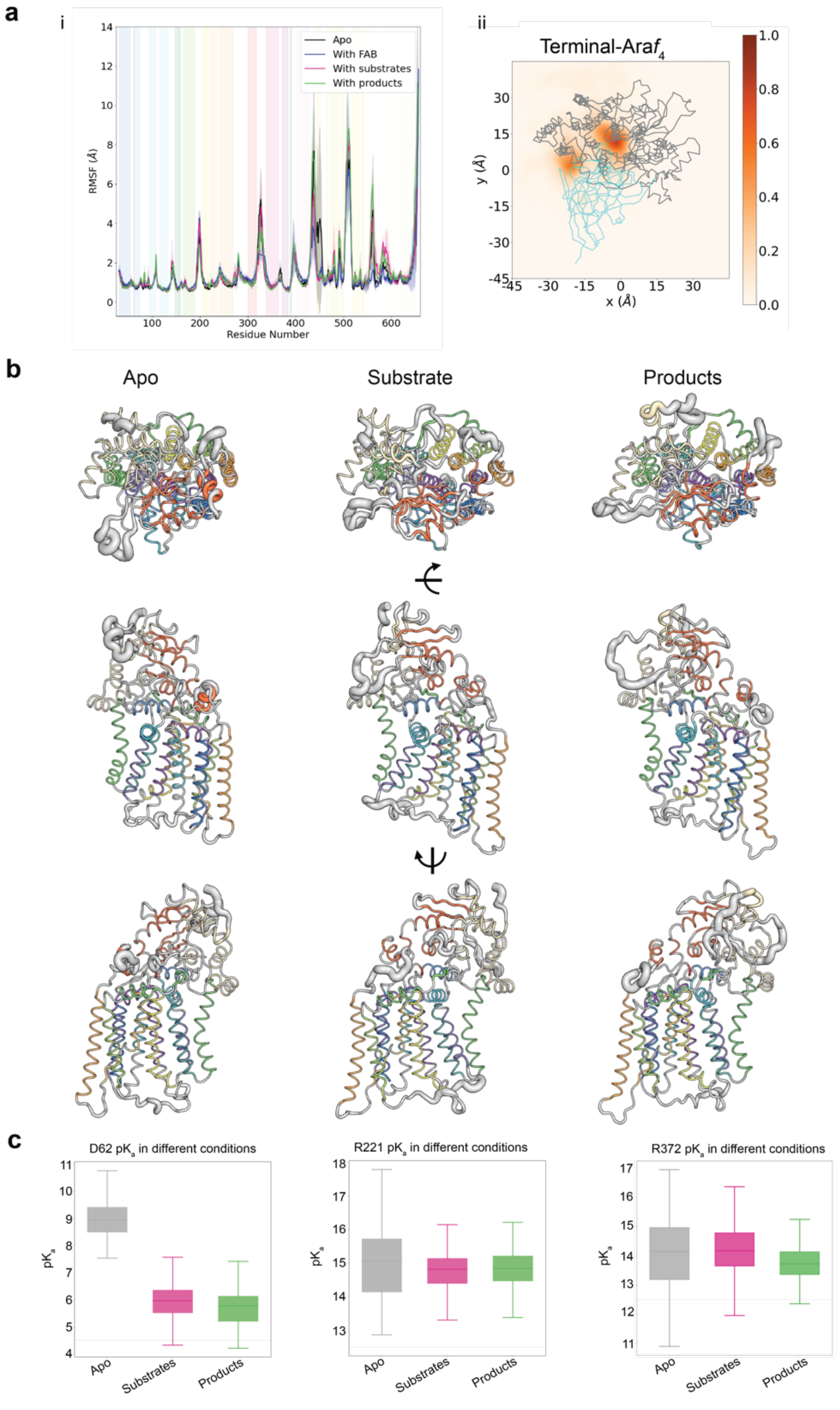

Supplementary Fig. 13 Conformational Dynamics and pK_a Analysis of AftB.

(a): (i) The root mean squared fluctuation (RMSF) of AftB in four states: apo, Fab-bound, substrate-bound, and product-bound. RMSF values per residue are averaged over three independent simulations (n=3), with the outline signifying the standard error. The different topological regions of the protein as shown in Fig. 1c are highlighted by color. (ii) Coarse-grained molecular dynamics (CG-MD) analysis plotting the density in the x and y dimensions of terminal-Ara*f*$_4$ relative to the protein shown in gray and Fab in cyan, where darker regions indicate higher density of terminal-Ara*f*$_4$ binding events. (b) The RMSF shown projected on the AftB structures, where the thicker regions are more dynamic in simulations. The helices are colored as in Fig. 1c. (c) Measured pK_a values of selected residues in various simulation conditions. Error bars represent standard error (n=3 independent simulations). Expected value for that residue is shown as a gray line. The first and third quartiles of the data are shown by the box bounds, with the median represented by the line within the box. The whiskers show the furthest data point within 1.5x of the interquartile range of the box, with outliers omitted for clarity.

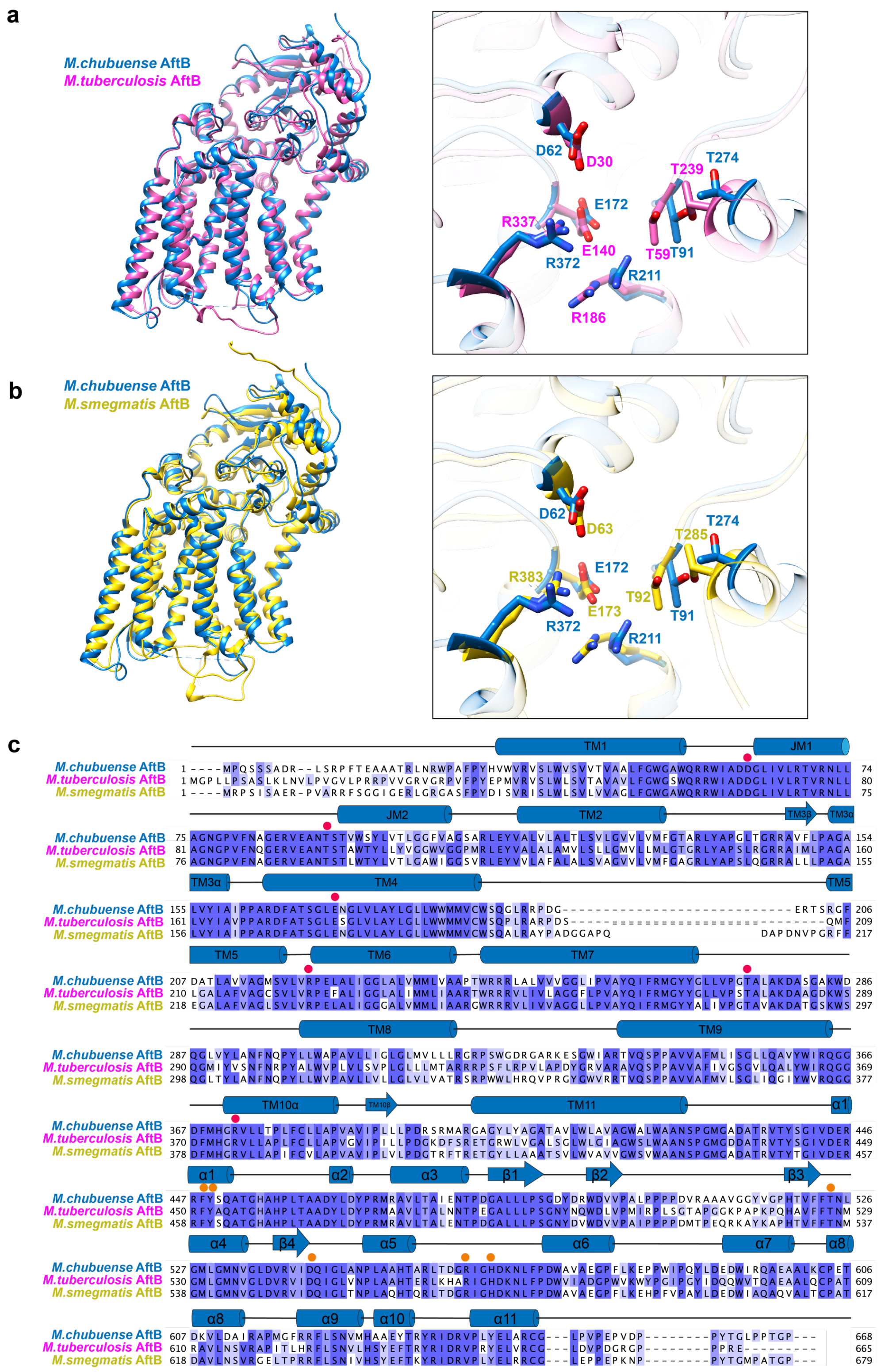

a
M.chubuense AftB
M.tuberculosis AftB
D62
D30
T239
T274
E172
R337
E140
T59
T91
R372
R211
R186
b
M.chubuense AftB
M.smegmatis AftB
D62
D63
T285
T274
E172
R383
T92
E173
T91
R372
R211
c
M.chubuense AftB
M.tuberculosis AftB
M.smegmatis AftB
TM1
JM1
JM2
TM2
TM3β
TM3α
TM4
TM5
TM6
TM7
TM8
TM9
TM10α
TM10β
TM11
α1
α2
α3
β1
β2
β3
α4
β4
α5
α6
α7
α8
α9
α10
α11

Supplementary Figure 14. Structural comparison of AftB orthologs and sequence conservation analysis.

(a,b) Structural superposition of *Mc*AftB (blue) with AlphaFold2 predictions of *M. tuberculosis* AftB (magenta, RMSD 2.8 Å across 606 pairs) (a) and *M. smegmatis* AftB (yellow, RMSD 2.4 Å across 617 pairs) (b). (c) Multiple sequence alignment of AftB from *M. chubuense*, *M. tuberculosis*, and *M. smegmatis*. Key residues for catalysis and donor substrate binding identified from the cryo-EM structure are marked with red dots and are strictly conserved across all three species. Key residues predicted to be involved in acceptor substrate engagement, including F448, Y449, T524, D541, R560, and H563, are marked with orange dots. Sequence identities between *Mc*AftB and *M. tuberculosis* AftB (74%) and *M. smegmatis* AftB (71%) demonstrate high conservation among mycobacterial AftB orthologs.

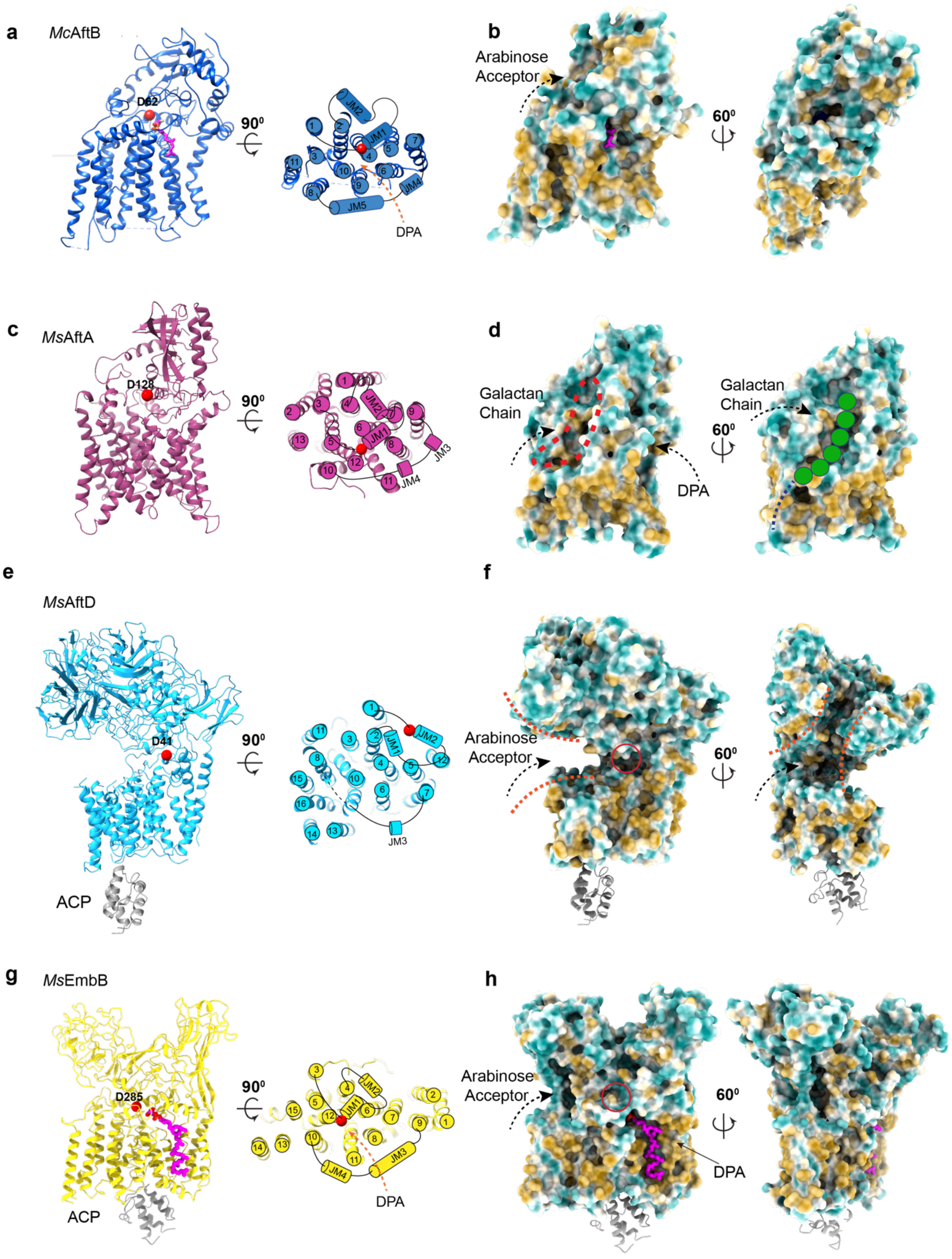

Supplementary Figure 15. Structural comparison of mycobacterial arabinosyltransferases.

(a,c,e,g) Cartoon representations of *Mc*AftB (2F-FPA-bound), *Mt*AftA (PDB: 8IF8), *Ms*AftD (PDB: 6W98), and *Ms*EmbB (DPA-bound) (PDB: 7BWR) shown in two orientations. Left panels show views parallel to the membrane plane; right panels show views perpendicular to the membrane plane from the periplasmic side. Catalytic aspartate residues (D62, D128, D41, and D285, respectively) are shown as red spheres. ACP is colored in grey for AftD and EmbB. 2F-FPA and DPA molecules are shown as magenta sticks. (b,d,f,h) Surface representations colored by hydrophobicity (cyan to orange) for corresponding proteins. In *Mc*AftB (b), the proposed arabinan acceptor entrance is indicated by a black dotted arrow. For *Mt*AftA (d), both the galactan chain acceptor and DPA substrate entrances are marked with black arrows, with the elongated cavity for galactan binding highlighted with dotted red lines. The *Ms*AftD structure (f) reveals the distinctive cleft architecture, with the proposed acceptor substrate entrance marked by a black dotted arrow. In *Ms*EmbB (h), the arabinan acceptor entrance is indicated by a black dotted arrow, with bound DPA shown in magenta.

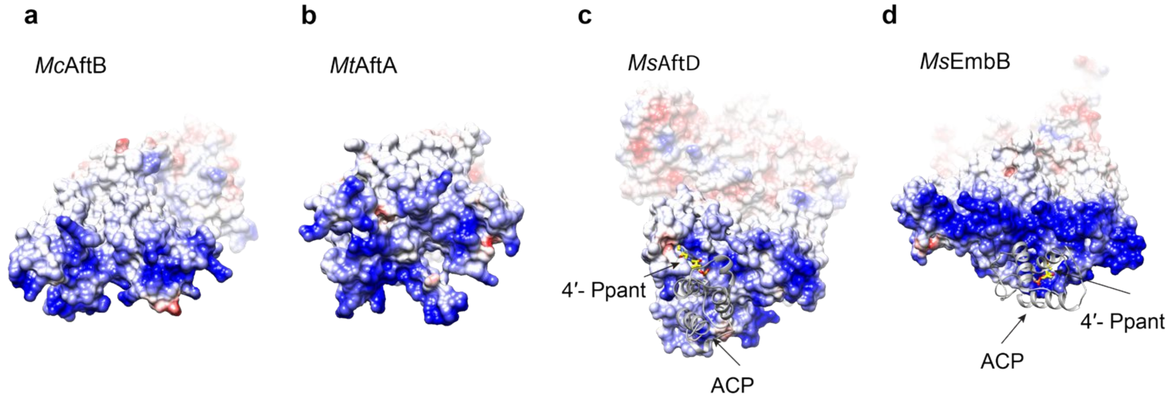

Supplementary Figure 16. Cytoplasmic surface comparison of mycobacterial arabinosyltransferases and ACP interactions.

Surface electrostatic potential representations viewed from from the cytoplasm for (a) *Mc*AftB, (b) *Mt*AftA, (c) *Ms*AftD with bound ACP, and (d) *Ms*EmbB with bound ACP. ACP is shown in grey cartoon representation, with its prosthetic group 4'-phosphopantetheine (4'-Ppant) shown in stick representation in panels c and d.

Supplementary Table 1. Cryo-EM data collection, refinement, and validation statistics.

	Apo AftB	AftB bound with 2F-FPA
**Data Collection**		
Microscope	FEI Titan Krios-CEC	FEI Titan Krios-NYSBC
Camera	Gatan K3	Gatan K3
Voltage (kV)	300	300
Electron expose (e-/Å2)	58	58
Defocus range (μm)	-1.2 to -2.2	-0.8 to -2.5
Pixel size (Å)	0.87	0.846
Symmetry imposed	C1	C1
Initial particle images (No.)	5,031,234	5,299,368
Final particle images (No.)	192,312	146,894
Final Resolution (Å)	2.85	3.40
FSC threshold	0.143	0.143
Map sharpening b-factor (Å2)	-97.0	-89.9
**Refinement**		
Model composition		
Non-hydrogen atoms	4741	4654
Protein residues	617	599
Ligands	0	1
Waters	0	0
Mean B factor (Å2)		
Protein	31.7	32.5
Ligands		45.9
R.m.s. deviation		
Bond lengths (Å)	0.003	0.003
Bond angles (°) Validation	0.51	0.49
Clashscore	2	8
Rotamers outlier (%)	0	0
Ramachandran plot		
Favored (%)	97	96.5
Allowed (%)	3	3.5
Disallowed (%)	0	0

Supplementary Table 2. Primers used for site-directed mutagenesis.

Mutant	Primer Sequences
D62A	Forward: GCCTGGCAGCGCCGCTGGATCGCCGACGCCGGCCTGATCGTGCTCCGCACGGTGCGA<br>Reverse: TCGCACCGTGCGGAGCACGATCAGGCCGGCGTCGGCGATCCAGCGGCGCTGCCAGGC
R221A	Forward: GCCGGAATGTCGGTGCTGGTGGCCCCCGAGCTCGCGCTGATCGGC<br>Reverse: GCCGATCAGCGCGAGCTCGGGGGCCACCAGCACCGACATTCCGGC
R221E	Forward: GTCGCCGGAATGTCGGTGCTGGTGGAACCCGAGCTCGCGCTGATCGGCGGC<br>Reverse: GCCGCCGATCAGCGCGAGCTCGGGTTCCACCAGCACCGACATTCCGGCGAC
R372A	Forward: GGCGGCGATTTCATGCACGGCGCGGTGTTGCTGACCCCGCTGTTC<br>Reverse: GAACAGCGGGGTCAGCAACACCGCGCCGTGCATGAAATCGCCGCC
R372E	Forward: GGCGGCGATTTCATGCACGGCGAGGTGTTGCTGACCCCGCTGTTC<br>Reverse: GAACAGCGGGGTCAGCAACACCTCGCCGTGCATGAAATCGCCGCC
F448A	Forward: TACAGCGGCATCGTCGACGAGCGCCGGGCCTACTCCCAGGCGACCGGTCACGCGCAC<br>Reverse: GTGCGCGTGACCGGTCGCCTGGGAGTAGGCCCGGCGCTCGTCGACGATGCCGCTGTA
F449A	Forward: AGCGGCATCGTCGACGAGCGCCGGTTCGCCTCCCAGGCGACCGGTCACGCGCACCCG<br>Reverse: CGGGTGCGCGTGACCGGTCGCCTGGGAGGCGAACCGGCGCTCGTCGACGATGCCGCT

SUPPLEMENTARY METHODS

Synthesis of Ara*f*4 and FPA.

General Methods

All reagents were purchased from commercial sources and used without further purification unless noted. Dichloromethane, *N*,*N*-dimethylformamide, tetrahydrofuran and toluene used in reactions were taken from a solvent purification system, in which the solvents were purified by successive passage through columns of alumina and copper under argon. Unless stated otherwise, all reactions were carried out in oven-dried round-bottom flasks and were performed under a positive pressure of argon. Reactions were monitored by thin-layer chromatography (TLC) on silica gel 60 F_{254} (0.25 mm; Merck) glass plates. TLC spots were detected under UV light and by charring with a solution of *p*-anisaldehyde (7.5 mL) in ethanol (350 mL), acetic acid (10 mL) and sulfuric acid (10 mL). In

reaction work-up involving extractions, solutions of organic solvents were washed with equal amounts of aqueous solutions. Organic solvents were removed under reduced pressure at 40 ºC on a rotary evaporator. All column chromatography was performed on silica gel 60 (4060 μm). Optical rotations were measured on a PerkinElmer 241 or 341 polarimeter at 22 ± 2 ºC at the sodium D line (589 nm) and are in units of (deg×mL)/(dm×g). ^1H NMR spectra were recorded at 500 or 600 MHz, and chemical shifts are referenced to residual $CHCl_3$ (7.26 ppm, $CDCl_3$); CD_2HOD (3.31 ppm, CD_3OD), or HOD (4.78 ppm, D_2O). ^{13}C NMR spectra were ^1H decoupled and were recorded at 126 or 150 MHz, and chemical shifts are referenced to internal $CDCl_3$ (77.06 ppm, $CDCl_3$), CD_3OD (49.15 ppm, CD_3OD), or external acetone (31.07 ppm, D_2O). ^{31}P NMR spectra were ^1H decoupled and were acquired at 202 MHz. Peak assignments were based on two-dimensional NMR (COSY, HSQC and HMBC) experiments. High-resolution electrospray ionization (ESI) and atmospheric pressure photoionization (APPI) mass spectrometry [3]spectra were recorded on an Agilent Technologies 6220 (Santa Clara, California, U.S.A.) time-of-flight (TOF) mass spectrometer or on a Waters LCT (Manchester, U.K.) TOF mass spectrometer with samples dissolved in an appropriate solvent.

Synthesis of Ara*f*$_4$

***p*-Tolyl 2-*O*-benzoyl-3,5-di-*O*-fluorenylmethoxycarbonyl-1-thio-α-D-arabinofuranoside (S2).** To a stirred solution of **S1**[3](10.78 g, 29.9 mmol) in CH_2Cl_2 (150 mL) at room temperature was added pyridine (23.73 g, 299.0 mmol) and 9-fluorenylmethoxycarbonyl chloride (19.34 g, 74.75 mmol). After stirring for 2 h, the reaction mixture was concentrated, dissolved in CH_2Cl_2 (75 mL) followed by washing with 1N HCl, water and brine. The organic layer was dried over Na_2SO_4, [3]filtered and concentrated to dryness. The crude residue was purified by flash chromatography (6:1 hexanes–EtOAc) to afford **S2** (22.01 g, 92%) as a colorless syrup: R_f = 0.26 (6:1 hexanes–EtOAc); $[\alpha]_D^{25}$ +56.0 (c = 2.33, $CHCl_3$); ^1H NMR (500 MHz, $CDCl_3$) δ 8.08 (dd, J = 8.4, 1.3 Hz, 2H, Ar), 7.80–7.72 (m, 4H, Ar), 7.66–7.61 (m, 2H, Ar), 7.59 (ddd, J = 7.6, 6.7, 0.9 Hz, 2H, Ar), 7.52 (ddt, J = 8.7, 7.1, 1.3 Hz, 1H, Ar), 7.47 (d, J = 8.1 Hz, 2H, Ar), 7.44–7.36 (m, 6H, Ar), 7.36–7.30 (m, 2H, Ar), 7.29–7.24 (m, 2H, Ar), 7.15–7.11 (m, 2H, Ar), 5.70 (dd, J = 1.7, 0.9 Hz, 1H, H-1), 5.65 (app t, J = 1.9, 1.9 Hz, 1H, H-2), 5.27 (ddd, J = 5.2, 2.0, 0.8 Hz, 1H, H-3), 4.74–4.68 (m, 1H, H-4), 4.61 (dd, J = 11.9, 3.5 Hz, 1H, H-5), 4.54 (dd, J = 11.9, 4.9 Hz, 1H, H-5), 4.49 (dd, J = 10.4, 7.5 Hz, 1H, Fmoc OCH_2CH), 4.43 (dd, J = 10.5, 7.5 Hz, 1H, Fmoc OCH_2CH), 4.38 (d, J = 7.7 Hz,

2H, Fmoc OCH_2CH), 4.30 (t, J = 7.5 Hz, 1H, Fmoc OCH_2CH), 4.22 (t, J = 7.6 Hz, 1H, Fmoc OCH_2CH), 2.32 (s, 3H, ArCH_3); ^{13}C NMR (126 MHz, $CDCl_3$) δ 165.4 (C=O), 155.1 (C=O), 154.5 (C=O), 143.4 (Ar), 143.4 (Ar), 143.3 (Ar), 143.2 (Ar), 141.5 (Ar), 141.5 (Ar), 141.4 (Ar), 138.4 (Ar), 133.8 (Ar), 133.1 (2 x Ar), 130.1 (2 x Ar), 130.0 (2 x Ar), 129.6 (Ar), 129.0 (Ar), 128.7 (2 x Ar), 128.1 (2 x Ar), 128.0 (2 x Ar), 127.4 (2 x Ar), 127.4 (2 x Ar), 127.3 (2 x Ar), 125.4 (Ar), 125.4 (2 x Ar), 120.3 (2 x Ar), 120.2 (2 x Ar), 91.5 (C-1), 81.8 (C-2), 80.7 (C-3), 80.1 (C-4), 70.8 (Fmoc O$C$$H_2$CH), 70.5 (Fmoc O$C$$H_2$CH), 66.4 (C-5), 46.8 (2 x Fmoc $OCH_2$$C$H), 21.3 (Ar$C$$H_3$); HRMS (ESI–TOF) m/z $[M + Na]^+$ calcd for $C_{49}H_{40}O_9NaS$ 827.2291, found 827.2287.

***p*-Tolyl 2,3-di-*O*-benzoyl-5-*O*-fluorenylmethoxycarbonyl-1-thio-α-D-arabinofuranoside (S4).** To a stirred solution of **S3**[3]2.32 g, 5.00 mmol) in CH_2Cl_2 (25 mL) at room temperature was added pyridine (3.95 g, 50.0 mmol) and 9-fluorenylmethoxycarbonyl chloride (1.94 g, 7.50 mmol). After stirring for 2 h, the reaction mixture was concentrated, dissolved in CH_2Cl_2 (50 mL) followed by washing with 1N HCl, water and brine. The organic layer was dried over Na_2SO_4, filtered and concentrated to dryness. The crude residue was purified by flash chromatography (6:1 hexanes–EtOAc) to afford **S4** (2.78 g, 81%) as a colorless syrup: R_f = 0.29 (6:1 hexanes–EtOAc); $[\alpha]_D^{25}$ +58.5 (c = 2.34, $CHCl_3$); ^{1}H NMR (500 MHz, $CDCl_3$) δ 8.17–8.07 (m, 4H, Ar), 7.76 (dd, J = 7.6, 1.0 Hz, 2H, Ar), 7.65–7.56 (m, 3H, Ar), 7.54–7.45 (m, 5H, Ar), 7.43–7.35 (m, 4H, Ar), 7.30–7.24 (m, 2H, Ar), 7.15–7.10 (m, 2H, Ar), 5.77 (s, 1H, H-1), 5.74 (d, J = 1.2 Hz, 1H, H-2), 5.59–5.54 (m, 1H, H-3), 4.81–4.75 (m, 1H, H-4), 4.69 (dd, J = 11.6, 3.5 Hz, 1H, H-5), 4.63 (dd, J = 11.9, 5.1 Hz, 1H, H-5), 4.38 (d, J = 7.6 Hz, 2H, Fmoc OCH_2CH), 4.23 (t, J = 7.6 Hz, 1H, Fmoc OCH_2CH), 2.32 (s, 3H, ArCH_3); ^{13}C NMR (126 MHz, $CDCl_3$) δ 165.8 (C=O), 165.4 (C=O), 155.2 (C=O), 143.5 (Ar), 143.5 (Ar), 141.4 (2 x Ar), 138.3 (Ar), 133.8 (Ar), 133.8 (Ar), 133.0 (2 x Ar), 130.2 (2 x Ar), 130.1 (2 x Ar), 130.0 (2 x Ar), 129.8 (Ar), 129.1 (Ar), 129.1 (Ar), 128.7 (2 x Ar), 128.7 (2 x Ar), 128.0 (2 x Ar), 127.4 (2 x Ar), 125.4 (2 x Ar), 120.2 (2 x Ar), 92.1 (C-1), 82.0 (C-2), 81.4 (C-4), 78.1(C-3), 70.5 (Fmoc O$C$$H_2$CH), 66.7 (C-5), 46.8 (Fmoc $OCH_2$$C$H), 21.3 (Ar$C$$H_3$); HRMS (ESI–TOF) m/z $[M + Na]^+$ calcd for $C_{41}H_{34}O_8NaS$ 709.1872, found 709.1871.

8-Azidooctyl 2-*O*-benzoyl-3,5-di-*O*-fluorenylmethyloxycarbonyl-α-D-arabinofuranosyl-(1→5)-2,3-di-*O*-benzoyl-α-D-arabinofuranoside (S6). Compound **S5**[4] (0.1 g, 0.19 mmol) and **S2** (0.17 g, 0.21 mmol) were dissolved in dry CH_2Cl_2 (2 mL). The solution was then mixed with

freshly activated 4 Å molecular sieves. The suspension was stirred at 0 °C for 15 min before NIS (70 mg, 1.5 equiv) and AgOTf (10 mg, 0.2 equiv) were added. The mixture was gradually warmed to room temperature and the reaction was monitored by TLC. When the reaction was complete, solid $Na_2S_2O_3$ was added until the solution became colourless and then and the mixture was filtered through Celite and concentrated *in vacuo*. The filtrate was redissolved CH_2Cl_2, extracted with water and the organic layer was concentrated. Column chromatography (20:1 Toluene–EtOAc) of the residue gave **S6** as colorless syrup (0.21 g, 94%). R_f = 0.44 (8:1 Toluene–EtOAc); $[\alpha]_D^{25}$ +4.5 (*c* 0.44, $CHCl_3$); ^{1}H NMR (600 MHz, $CDCl_3$, δ_H) 8.13–8.07 (m, 6 H, Ar), 7.76–7.74 (m, 2 H, Ar, 7.69–7.66 (m, 2 H, Ar), 7.59–7.49 (m, 5 H, Ar), 7.46–7.42 (m, 6 H, Ar), 7.39–7.29 (m, 6 H, Ar), 7.27–7.24 (m, 2 H, Ar), 7.20–7.17 (m, 2 H, Ar), 5.71 (d, 1 H, *J* = 5.0 Hz, H-3), 5.63 (d, 1 H, *J* = 1.2 Hz, H-2'), 5.52 (d, 1 H, *J* = 1.3 Hz, H-2), 5.46 (s, 1 H, H-1'), 5.26 (s, 1 H, H-1), 5.19 (dd, 1 H, *J* = 4.6, 1.2 Hz, H-3'), 4.71-4.67 (m, 2H, H-4', H-5'), 4.53 (dd, 1 H, *J* = 11.5, 4.4 Hz, H-5'), 4.43 (ddd, 1 H, *J* = 4.4, 4.6, 2.9 Hz, H-4), 4.36 (d, 2H, *J* = 7.7 Hz, Fmoc OC$\underline{H}_2$), 4.27—4.25 (m, 3H, H-5, Fmoc OC$\underline{H}_2$), 4.20 (t, 1H, *J* = 7.6 Hz, Fmoc C$\underline{H}$), 4.07 (t, 1H, *J* = 7.4 Hz, Fmoc C$\underline{H}$), 3.95 (dd, 1 H, *J* = 11.1, 2.6 Hz, H-5), 3.78 (ddd, 1 H, *J* = 9.5, 6.6, 6.6 Hz, octyl OC$\underline{H}_2$), 3.54 (ddd, 1 H, *J* = 9.5, 6.3, 6.3 Hz, octyl OC$\underline{H}_2$), 3.21 (dd, 2 H, *J* = 7.0, 7.0 Hz, octyl C$\underline{H}_2N_3$), 1.66–1.54 (m, 4 H, octyl C$\underline{H}_2$), 1.40–1.26 (m, 8 H, octyl C$\underline{H}_2$); ^{13}C NMR (150 MHz, $CDCl_3$, δ_C) 165.8 (C=O), 165.6 (C=O), 165.2 (C=O), 155.1 (C=O), 154.6 (C=O), 143.3 (Ar × 2), 143.1 (Ar × 2), 141.3 (Ar × 2), 141.2 (Ar × 2), 133.6 (Ar), 133.5 (Ar × 2), 130.0 (Ar × 4), 129.9 (Ar × 2), 129.4 (Ar), 129.3 (Ar), 129.1 (Ar), 128.6 (Ar × 2), 128.5 (Ar × 4), 127.9 (Ar × 2), 127.8 (Ar × 2), 127.2 (Ar × 3), 127.1 (Ar), 125.3 (Ar), 125.2 (Ar), 125.1 (Ar × 2), 120.0 (Ar × 4), 105.5 (C-1), 105.4 (C-1'), 82.0 (2C, C-2, C-4), 81.1 (C-2'), 80.9 (C-4'), 80.5 (C-3'), 77.3 (C-3), 70.4 (Fmoc O$\underline{C}H_2$), 70.3 (Fmoc O$\underline{C}H_2$), 67.4 (octyl OCH_2), 66.7 (C-5'), 65.7 (C-5), 51.4 (octyl $\underline{C}H_2N_3$), 46.5 (Fmoc $\underline{C}$H), 46.6 (Fmoc $\underline{C}$H), 29.5 (octyl CH_2), 29.3 (octyl CH_2), 29.1 (octyl CH_2), 28.8 (octyl CH_2), 26.7 (octyl CH_2), 26.1 (octyl CH_2). ESIMS m/z calcd. for (M + Na^+) $C_{69}H_{65}NaN_3O_{16}$: 1214.4257. Found: 1214.4267.

8-Azidooctyl 2-*O*-benzoyl-α-D-arabinofuranosyl-(1→5)-2,3-di-*O*-benzoyl-α-D-arabinofuranoside (S7). To a solution of compound **S6** (2.43 g, 2.03 mmol) and EtOAc (20.0 mL) was added piperidine (5.0 mL) at 25 °C. The reaction mixture was stirred at room temperature for 8 h and then water was added followed by 1N HCl until the solution was neutral

by pH paper. The organic layer was separated, dried with $MgSO_4$, filtered, and concentrated. The product was purified by chromatography (3:1, hexane–EtOAc) to yield **S7** (1.0 g, 67%) as a colorless syrup. R_f = 0.49 (1:1 hexane–EtOAc); $[\alpha]_D^{25}$ +49.7 (*c* 0.97, $CHCl_3$); ^{1}H NMR (600 MHz, $CDCl_3$, δ_H) 8.08–8.06 (m, 4 H, Ar), 8.00 (dd, 2 H, *J* = 10.0, 1.6 Hz, Ar), 7.62–7.56 (m, 3 H, Ar), 7.47–7.43 (m, 6 H, Ar), 5.54 (dd, 1 H, *J* = 5.9, 1.8 Hz, H-3), 5.51 (d, 1 H, *J* = 1.8 Hz, H-2), 5.39 (s, 1 H, H-1'), 5.26 (s, 1 H, H-1), 5.16 (d, 1 H, *J* = 3.1 Hz, H-2'), 4.41 (ddd, 1H, *J* = 5.7, 5.7, 4.1 Hz, H-4), 4.53 (ddd, 1 H, *J* = 6.6, 6.5, 3.8 Hz, H-4'), 4.19–4.14 (m, 2 H, H-3', H-5), 3.97–3.92 (m, 2 H, H-5, H-5'), 3.80–3.74 (m, 2 H, H-5', octyl $OC\underline{H}_2$), 3.53 (ddd, 1 H, *J* = 9.5, 6.6, 6.6 Hz, octyl $OC\underline{H}_2$), 3.46 (d, 1H, *J* = 7.1 Hz, 3-OH), 3.23 (dd, 2 H, *J* = 8.4, 8.4 Hz, octyl $C\underline{H}_2N_3$), 2.00 (dd, 1 H, *J* = 5.8, 3.4 Hz, 5-OH), 1.68–1.54 (m, 4 H, octyl $C\underline{H}_2$), 1.45–1.26 (m, 8 H, octyl $C\underline{H}_2$); ^{13}C NMR (150 MHz, $CDCl_3$, δ_C) 166.6 (C=O), 165.9 (C=O), 165.5 (C=O), 133.6 (Ar), 133.5 (Ar × 2), 130.0 (Ar × 2), 129.9 (Ar × 2), 129.8 (Ar × 2), 129.2 (Ar), 129.1 (Ar), 129.0 (Ar), 128.6 (Ar × 4), 128.5 (Ar × 2), 105.6 (C-1), 105.1 (C-1'), 86.0 (C-2'), 84.5 (C-4'), 81.9 (C-2), 81.7 (C-4), 77.4 (C-3), 76.6 (C-3'), 67.5 (octyl OCH_2), 65.8 (C-5), 62.2 (C-5'), 51.4 (octyl $\underline{C}H_2N_3$), 29.5 (octyl CH_2), 29.3 (octyl CH_2), 29.1 (octyl CH_2), 28.8 (octyl CH_2), 26.7 (octyl CH_2), 26.1 (octyl CH_2). ESIMS *m/z* calcd. for (M + Na^+) $C_{39}H_{45}NaN_3O_{12}$: 770.2896. Found: 770.2901.

8-Azidooctyl 2,3-di-*O*-benzoyl-5-*O*-fluorenylmethyloxycarbonyl-α-D-arabinofuranosyl-(1→5)-[2,3-di-*O*-benzoyl-5-*O*-fluorenylmethyloxycarbonyl-α-D-arabinofuranosyl-(1→3)]-2-*O*-benzoyl-α-D-arabinofuranosyl-2,3-di-*O*-benzoyl-α-D-arabinofuranoside (S8). Compound **S7** (0.99 g, 1.32 mmol) and **S4** (1.91 g, 2.78 mmol) were dissolved in dry CH_2Cl_2 (20 mL). The solution was then mixed with freshly activated 4 Å molecular sieves and the suspension was stirred in 0 °C for 15 min before NIS (445 mg, 1.5 equiv) and AgOTf (68 mg, 0.2 equiv) were added. The mixture was warmed to room temperature and the reaction was monitored by TLC. When the reaction was complete, solid $Na_2S_2O_3$ was added until the solution became colourless and then and the mixture was filtered through Celite and concentrated *in vacuo*. The filtrate was redissolved CH_2Cl_2, extracted with water and the organic layer was concentrated. Column chromatography of the residue (6:1:2 hexane–EtOAc–CH_2Cl_2) gave **S8** as a colorless syrup (1.60 g, 65%). R_f = 0.83 (4:1:2 hexane–EtOAc–CH_2Cl_2); $[\alpha]_D^{25}$ +10.2 (*c* 1.9, $CHCl_3$); ^{1}H NMR (600 MHz, $CDCl_3$, δ_H) 8.08–7.96 (m, 10 H, Ar), 7.93–7.91 (m, 4 H, Ar), 7.73–7.69 (m, 4 H, Ar),

7.55–7.32 (m, 26 H, Ar), 7.30–7.19 (m, 7 H, Ar), 5.61 (dd, 1 H, *J* = 4.8, 1.5 Hz), 5.56 (d, 1 H, *J* = 1.0 Hz), 5.54 (s, 1 H), 5.50 (m, 2 H), 5.45 (d, 1 H, *J* = 1.5 Hz), 5.40–5.39 (m, 3 H), 5.38 (dd, 1 H, *J* = 4.9, 1.4 Hz), 5.21 (s, 1H), 4.64 (dd, 1 H, *J* = 11.5, 3.1 Hz), 4.59–4.47 (m, 7H), 4.40 (ddd, 1 H, *J* = 4.8, 4.8, 3.0 Hz), 4.31 (d, 1H, *J* = 1.9 Hz), 4.30 (d, 1H, *J* = 1.3 Hz), 4.25 (d, 1H, *J* = 1.2 Hz), 4.23 (s, 1H), 4.28–4.08 (m, 4 H), 3.92–3.90 (m, 2 H), 3.75 (ddd, 1 H, *J* = 9.5, 6.6, 6.6 Hz, octyl OC$\underline{H}_2$), 3.49 (ddd, 1 H, *J* = 9.5, 6.2, 6.2 Hz, octyl OC$\underline{H}_2$), 3.20 (dd, 2 H, *J* = 7.0, 7.0 Hz, octyl C$\underline{H}_2N_3$), 1.63–1.52 (m, 4 H, octyl C$\underline{H}_2$), 1.40–1.26 (m, 8 H, octyl C$\underline{H}_2$); ^{13}C NMR (150 MHz, $CDCl_3$, δ_C) 165.7 (C=O × 2), 165.6 (C=O), 165.5 (C=O), 165.4 (C=O × 2), 164.8 (C=O), 155.1 (C=O), 154.9 (C=O), 143.4 (Ar × 4), 141.2 (Ar × 4), 133.5 (Ar × 3), 133.4 (Ar × 4), 130.0 (Ar × 3), 129.9 (Ar × 4), 129.8 (Ar × 7), 129.5 (Ar), 129.3 (Ar), 129.2 (Ar × 2), 129.1 (Ar), 129.0 (Ar), 128.9 (Ar), 128.6 (Ar × 2), 128.5 (Ar × 4), 128.4 (Ar × 3), 128.3 (Ar × 5), 127.8 (Ar × 4), 127.2 (Ar × 4), 125.3 (Ar × 2), 125.2 (Ar × 2), 120.0 (Ar × 4), 105.9 (C-1), 105.7 (C-1), 105.6 (C-1), 105.5 (C-1), 82.8, 82.0, 81.9, 81.6 (C × 2), 81.5, 81.3, 81.1, 80.9, 77.6, 77.2 (C × 2), 70.2 (Fmoc O$\underline{C}H_2$), 70.1 (Fmoc O$\underline{C}H_2$), 67.4 (octyl OCH_2), 67.4 (octyl O$\underline{C}H_2$), 66.9, 66.7, 65.8, 65.4, 51.4 (octyl $\underline{C}H_2N_3$), 46.7 (Fmoc $\underline{C}$H), 46.6 (Fmoc $\underline{C}$H), 29.5 (octyl CH_2), 29.3 (octyl CH_2), 29.1 (octyl CH_2), 28.8 (octyl CH_2), 26.7 (octyl CH_2), 26.1 (octyl CH_2). ESIMS *m/z* calcd. for (M + Na^+) $C_{107}H_{97}NaN_3O_{28}$: 1894.6151. Found: 1894.6143.

8-Azidooctyl α-D-arabinofuranosyl-(1→5)-[α-D-arabinofuranosyl-(1→3)]-α-D-arabinofuranosyl-2α-D-arabinofuranoside (Ara*f*$_4$). To a solution of compound **S8** (1.1 g, 0.58 mmol) in EtOAc (5.0 mL) was added piperidine (1.0 mL) at 25 °C. The reaction mixture was stirred at room temperature for 8 h and then water was added followed by 1N HCl until the solution was neutral by pH paper. The organic layer was separated, dried with $MgSO_4$, filtered, concentrated and then the residue was dissolved in methanol. Solid sodium methoxide (3 mg, 0.06 mmol) was added to the solution and the pH of the reaction mixture was checked (by pH paper) that it was basic. The mixture was then stirred for 16 h before Amberlite ® H^+ resin was used until the solution was neutral. The solution was concentrated and the residue was purified by chromatography (1:6, MeOH–EtOAc) to give **Ara*f*$_4$** (**V**, 0.21 g, 52%) as a colorless syrup. R_f = 0.3 (6:1:1 EtOAc–MeOH–H_2O); $[\alpha]_D^{25}$ +209.4 (*c* 0.36, MeOH); ^{1}H NMR (600 MHz, D_2O, δ_H) 5.14 (d, 1H, *J* = 1.5 Hz, H-1), 5.09 (s, 1 H, H-1), 5.07 (d, 1 H, *J* = 1.5 Hz, H-1), 4.99 (d, 1 H, *J* = 2.2 Hz, H-1), 4.30 (ddd, 1H, *J* =5.5 Hz, 5.5 Hz, 2.9 Hz), 4.27 (dd, 1 H, *J* = 2.1, 1.1 Hz), 4.15–

4.07 (m, 5 H), 4.04–3.99 (m, 3 H), 3.95–3.92 (m, 3H), 3.87–3.77 (m, 5H), 3.75–3.69 (m, 3 H), 3.57 (ddd, 1 H, *J* = 9.5, 6.3, 6.3 Hz, octyl OC<u>H</u>$_2$), 3.31 (dd, 2 H, *J* = 7.0, 7.0 Hz, octyl C<u>H</u>$_2$N$_3$), 1.62–1.57 (m, 4 H, octyl C<u>H</u>$_2$), 1.38–1.32 (m, 8 H, octyl C<u>H</u>$_2$); ^{13}C NMR (150 MHz, D$_2$O, δ_C) 107.4 (C-1), 107.3 (C-1), 107.2 (C-1), 107.1 (C-1), 84.0, 83.9, 82.4, 81.7, 81.5, 81.2, 80.9, 80.8, 79.1, 76.5, 76.4, 76.3, 68.6 (octyl OCH$_2$), 66.4, 66.3, 61.1 (C× 2), 51.2 (octyl CH$_2$N$_3$), 28.5 (octyl CH$_2$), 28.2 (octyl CH$_2$), 28.1 (octyl CH$_2$), 27.9 (octyl CH$_2$), 25.8 (octyl CH$_2$), 25.0 (octyl CH$_2$). ESIMS *m/z* calcd. for (M + Na$^+$) $C_{42}H_{43}NaN_3O_9$: 722.2954. Found: 722.2947.

Synthesis of FPA

***p*-Tolyl 2,3,5-tri-*O*-*tert*-butyldiphenylsilyl-1-thio-α-D-arabinofuranoside (S10).** To a solution of **S9**[5] (280 mg, 1.09 mmol) in dry DMF (5 mL) was added imidazole (1.34 g, 19.6 mmol), followed by TBDPSCl (1.68 mL, 6.55 mmol). The reaction mixture was stirred at 50 ºC for 12 h. After cooling to rt, excess TBDPSCl was quenched by the addition of ice-cold water and the solution was extracted with EtOAc. The organic layer was washed with H$_2$O and brine, dried with MgSO$_4$, filtered, and the filtrate was concentrated. The crude residue was purified by column chromatography (40:1 hexanes–EtOAc) to yield **S10** (1.04 g, 98%) as a colorless oil. R_f 0.65 (10:1 hexanes–EtOAc); $[\alpha]_D$ +24 (*c* 0.72, CHCl$_3$); ^{1}H NMR (500 MHz, CDCl$_3$, δ): 7.66–7.62 (m, 2 H, ArH), 7.59–7.52 (m, 6 H, ArH), 7.49–7.20 (m, 22 H, ArH), 7.15–7.12 (m, 2 H, ArH), 7.02–6.98 (m, 2 H, ArH), 5.25 (s, 1 H, H-1), 4.51 (ddd, $J_{4,5a}$ = 6.5 Hz, $J_{4,5b}$ = 6.0 Hz, $J_{3,4}$ = 2.1 Hz, 1 H, H-4), 4.43 (s, 1 H, H-2), 4.27 (d, $J_{3,4}$ = 2.1 Hz, 1 H, H-3), 3.59 (dd, $J_{5a,5b}$ = 10.5 Hz, $J_{4,5a}$ = 6.5 Hz, 1 H, H-5a), 3.52 (dd, $J_{5a,5b}$ = 10.5 Hz, $J_{4,5b}$ = 6.0 Hz, 1 H, H-5b), 2.30 (s, 3 H, ArC<u>H</u>$_3$), 1.01 (s, 9 H, SiC(CH$_3$)$_3$), 0.95 (s, 9 H, SiC(CH$_3$)$_3$), 0.83 (s, 9 H, SiC(CH$_3$)$_3$); ^{13}C NMR (126 MHz, CDCl$_3$, δ): 136.5 (Ar), 136.0 (Ar), 135.9 (Ar), 135.77 (Ar), 135.75 (Ar), 135.62 (Ar), 135.58 (Ar), 133.7 (Ar), 133.48 (Ar), 133.45 (Ar), 133.1 (Ar), 132.91 (Ar), 132.89 (Ar), 132.6 (Ar), 131.6 (Ar), 129.78 (Ar), 129.75 (Ar), 129.71 (Ar), 129.68 (Ar), 129.5 (Ar), 129.44 (Ar), 129.41 (Ar), 127.71 (Ar), 127.67 (Ar), 127.63 (Ar), 127.57 (Ar), 127.5 (Ar), 95.2 (C-1), 88.1 (C-4), 84.9 (C-2), 80.2 (C-3), 64.4 (C-5), 26.83 (SiC(<u>C</u>H$_3$)$_3$), 26.81 (SiC(<u>C</u>H$_3$)$_3$), 26.7 (SiC(<u>C</u>H$_3$)$_3$), 21.1 (Ar<u>C</u>H$_3$), 19.23 (Si<u>C</u>(CH$_3$)$_3$), 19.18 (Si<u>C</u>(CH$_3$)$_3$), 18.9 (Si<u>C</u>(CH$_3$)$_3$); HRMS–ESI–TOF (*m/z*): $[M+NH_4]^+$ calcd for $C_{60}H_{74}NO_4SSi_3$, 988.4641; found, 988.4631.

Dibenzyl (2,3,5-tri-*O*-*tert*-butyldiphenylsilyl-β-D-arabinofuranosyl) phosphate (S11). To a stirred solution of **S10** (255 mg, 0.263 mmol) in CH_2Cl_2 (3 mL) was added Br_2 (17 µL, 0.34 mmol). The reaction mixture was stirred at rt for 1 h before being concentrated. The crude glycosyl bromide was azeotropically dried with toluene and then used immediately. To a stirred solution of azeotropically dried dibenzyl phosphate (146 mg, 0.525 mmol) in toluene (1 mL) were added powdered 4 Å molecular sieves (250 mg) and Et_3N (95 µL, 0.68 mmol). The mixture was cooled to 0 ºC, and a solution of the glycosyl bromide in toluene (1 mL) was added slowly via a cannula. The transfer was completed by rinsing the flask twice with toluene (2 × 0.5 mL). The reaction mixture was warmed slowly to rt and stirred for 15 h before being filtered through a pad of Celite®, rinsed with EtOAc, and the filtrate was concentrated. The crude residue was purified by column chromatography (10% EtOAc–hexanes, containing 0.5% Et_3N) to yield **S11** (185 mg, 62% over two steps, β:α > 19:1) as a colorless oil. R_f 0.39 (5:1 hexanes–EtOAc); ^{1}H NMR (500 MHz, $CDCl_3$, δ): 7.60–7.51 (m, 9 H, ArH), 7.50–7.44 (m, 4 H, ArH), 7.42–7.15 (m, 27 H, ArH), 5.80 (dd, $J_{1,P}$ = 4.9 Hz, $J_{1,2}$ = 3.1 Hz, 1 H, H-1), 5.01 (dd, J_{gem} = 11.8 Hz, $J_{H,P}$ = 6.9 Hz, 1 H, OC<u>H</u>$_2$Ph), 4.90 (dd, J_{gem} = 11.8 Hz, $J_{H,P}$ = 7.7 Hz, 1 H, OC<u>H</u>$_2$Ph), 4.83–4.78 (m, 2 H, OC<u>H</u>$_2$Ph), 4.35 (dd, $J_{2,3}$ = 1.8 Hz, $J_{3,4}$ = 1.6 Hz, 1 H, H-3), 4.27 (ddd, $J_{4,5a}$ = 7.2 Hz, $J_{4,5b}$ = 6.8 Hz, $J_{3,4}$ = 1.6 Hz, 1 H, H-4), 4.17–4.14 (m, 1 H, H-2), 3.75 (dd, $J_{5a,5b}$ = 10.4 Hz, $J_{4,5a}$ = 7.2 Hz, 1 H, H-5a), 3.64 (dd, $J_{5a,5b}$ = 10.4 Hz, $J_{4,5b}$ = 6.8 Hz, 1 H, H-5b), 0.96 (s, 9 H, $SiC(CH_3)_3$), 0.95 (s, 9 H, $SiC(CH_3)_3$), 0.90 (s, 9 H, $SiC(CH_3)_3$); ^{13}C NMR (176 MHz, $CDCl_3$, δ): 136.0 (Ar), 135.8 (Ar), 135.7 (Ar), 135.5 (Ar), 133.5 (Ar), 133.3 (Ar), 133.0 (Ar), 132.65 (Ar), 132.61 (Ar), 132.59 (Ar), 129.8 (Ar), 129.72 (Ar), 129.67 (Ar), 129.54 (Ar), 129.51 (Ar), 128.33 (Ar), 128.31 (Ar), 128.15 (Ar), 128.12 (Ar), 127.8 (Ar), 127.69 (Ar), 127.68 (Ar), 127.66 (Ar), 127.64 (Ar), 127.60 (Ar), 127.58 (Ar), 127.5 (Ar), 100.8 (d, $J_{C,P}$ = 5.8 Hz, C-1), 86.8 (C-4), 78.1 (C-3), 78.0 (d, $J_{C,P}$ = 8.5 Hz, C-2), 68.9 (d, $J_{C,P}$ = 5.3 Hz, O<u>C</u>H$_2$Ph), 68.8 (d, $J_{C,P}$ = 5.3 Hz, O<u>C</u>H$_2$Ph), 64.5 (C-5), 26.85 (SiC(<u>C</u>H$_3$)$_3$), 26.82 (SiC(<u>C</u>H$_3$)$_3$), 26.7 (SiC(<u>C</u>H$_3$)$_3$), 19.3 (Si<u>C</u>(CH$_3$)$_3$), 19.09 (Si<u>C</u>(CH$_3$)$_3$), 19.07 (Si<u>C</u>(CH$_3$)$_3$); ^{31}P NMR (202 MHz, $CDCl_3$, δ): –0.45; HRMS–ESI–TOF (*m*/*z*): $[M+NH_4]^+$ calcd for $C_{67}H_{81}NO_8PSi_3$, 1142.5002; found, 1142.5002.

(*Z*,*Z*)-Farnesylphosphoryl-β-D-arabinofuranose (FPA). To a stirred solution of **S11** (182 mg, 0.162 mmol) in 10% EtOH–EtOAc (5.8 mL) were added Et_3N (560 µL, 4.04 mmol) and 5% palladium on carbon (344 mg, 0.162 mmol). The reaction vessel was purged with argon and then

equipped with a hydrogen-filled balloon. The reaction mixture was stirred at rt for 16 h before being filtered through a pad of Celite® with 10% EtOH–EtOAc, and the filtrate was concentrated. The crude arabinofuranosyl phosphate (163 mg, 0.142 mmol) and (*Z*,*Z*)-farnesol[6] (126 mg, 0.568 mmol) were azeotropically dried with toluene. The mixture was dissolved in pyridine (1.9 mL) and Cl_3CCN (142 µL, 1.42 mmol) was added. The resulting solution was stirred for 14 h at 55 ºC before being cooled to rt and concentrated. The crude phosphodiester was dissolved in a 15% solution of concentrated NH_4OH in CH_3OH (2.8 mL), and NH_4F (158 mg, 4.26 mmol) was added. After stirring for 15 h at 55 ºC, the reaction mixture was cooled to rt, and CH_2Cl_2 (4 mL) was added to precipitate any remaining NH_4F. The solution was filtered through a pad of Celite® and the filtrate was concentrated to a crude residue that was purified by column chromatography (gradient of 50%→70% CH_3OH–EtOAc). Residual colored impurities were removed by the addition of activated charcoal to the product in CH_3OH, followed by the filtration through a syringe filter (0.45 µm). The filtrate was concentrated to give **FPA** (**V,** 53.0 mg, 73% over three steps, β:α > 19:1) as a colorless oil. R_f 0.29 (4:25:71 H_2O–CH_3OH–CH_2Cl_2); ^{1}H NMR (500 MHz, CD_3OD, δ): 5.49 (dd, $J_{1,P}$ = 4.6 Hz, $J_{1,2}$ = 4.4 Hz, 1 H, H-1), 5.45–5.39 (m, 1 H, $OCH_2C\underline{H}$=C), 5.17–5.10 (m, 2 H, 2 × $CH_2C\underline{H}$=C), 4.48–4.39 (m, 2 H, $OC\underline{H}_2CH$=C), 4.07 (dd, $J_{2,3}$ = 8.1 Hz, $J_{3,4}$ = 6.9 Hz, 1 H, H-3), 3.98 (ddd, $J_{2,3}$ = 8.1 Hz, $J_{1,2}$ = 4.4 Hz, $J_{2,P}$ = 2.2 Hz, 1 H, H-2), 3.80 (ddd, $J_{3,4}$ = 6.9 Hz, $J_{4,5b}$ = 6.2 Hz, $J_{4,5a}$ = 3.2 Hz, 1 H, H-4), 3.75 (dd, $J_{5a,5b}$ = 12.0 Hz, $J_{4,5a}$ = 3.2 Hz, 1 H, H-5a), 3.63 (dd, $J_{5a,5b}$ = 12.0 Hz, $J_{4,5b}$ = 6.2 Hz, 1 H, H-5b), 2.15–1.99 (m, 8 H, 4 × allylic CH_2), 1.74 (s, 3 H, CH_3), 1.68 (s, 6 H, 2 × CH_3), 1.61 (s, 3 H, CH_3); ^{13}C NMR (126 MHz, CD_3OD, δ): 141.0 (CH=$\underline{C}$), 136.7 (CH=$\underline{C}$), 132.5 (CH=$\underline{C}$), 125.9 ($\underline{C}$H=C), 125.5 ($\underline{C}$H=C), 123.4 (d, $J_{C,P}$ = 8.3 Hz, $\underline{C}$H=C), 99.2 (d, $J_{C,P}$ = 6.1 Hz, C-1), 85.1 (C-4), 79.4 (d, $J_{C,P}$ = 7.7 Hz, C-2), 75.4 (C-3), 64.3 (C-5), 63.5 (d, $J_{C,P}$ = 5.4 Hz, $O\underline{C}H_2CH$=C), 33.4 (allylic CH_2), 33.1 (allylic CH_2), 27.8 (allylic CH_2), 27.7 (allylic CH_2), 26.1 (CH_3), 23.9 (CH_3), 23.8 (CH_3), 17.9 (CH_3); ^{31}P NMR (202 MHz, CD_3OD, δ): 0.31; HRMS–ESI–TOF (*m*/*z*): $[M-H]^-$ calcd for $C_{20}H_{34}O_8P$, 433.1997; found, 433.1996.

SUPPLEMENTARY REFERENCES

1. Krishna, R. *et al.* Generalized biomolecular modeling and design with RoseTTAFold All-Atom. *Science (1979)* **384**, (2024).
2. Kyte, J. & Doolittle, R. F. A simple method for displaying the hydropathic character of a protein. *J Mol Biol* **157**, 105–132 (1982).

3. Cociorva, O. M. & Lowary, T. L. Synthesis of oligosaccharides as potential inhibitors of mycobacterial arabinosyltransferases. Di- and trisaccharides containing C-5 modified arabinofuranosyl residues. *Carbohydr Res* **339**, 853–865 (2004).
4. Joe, M., Bai, Y., Nacario, R. C. & Lowary, T. L. Synthesis of the docosanasaccharide arabinan domain of mycobacterial arabinogalactan and a proposed octadecasaccharide biosynthetic precursor. *J Am Chem Soc* **129**, 9885–9901 (2007).
5. D'Souza, F. W., Ayers, J. D., McCarren, P. R. & Lowary, T. L. Arabinofuranosyl oligosaccharides from mycobacteria: Synthesis and effect of glycosylation on ring conformation and hydroxymethyl group rotamer populations. *J Am Chem Soc* **122**, 1251–1260 (2000).
6. Snyder, S. A., Treitler, D. S. & Brucks, A. P. Simple reagents for direct halonium-induced polyene cyclizations. *J Am Chem Soc* **132**, 14303–14314 (2010).
